# Structure of HI-6•Sarin-Acetylcholinesterase Determined by X-Ray Crystallography and Molecular Dynamics Simulation: Reactivator Mechanism and Design

**DOI:** 10.1371/journal.pone.0005957

**Published:** 2009-06-18

**Authors:** Fredrik Ekström, Andreas Hörnberg, Elisabet Artursson, Lars-Gunnar Hammarström, Gunter Schneider, Yuan-Ping Pang

**Affiliations:** 1 Swedish Defence Research Agency, CBRN Defence and Security, Umeå, Sweden; 2 Department of Medical Biochemistry and Biophysics, Karolinska Institutet, Stockholm, Sweden; 3 Computer-Aided Molecular Design Laboratory, Mayo Clinic, Rochester, Minnesota, United States of America; Weizmann Institute of Science, Israel

## Abstract

Organophosphonates such as isopropyl metylphosphonofluoridate (sarin) are extremely toxic as they phosphonylate the catalytic serine residue of acetylcholinesterase (AChE), an enzyme essential to humans and other species. Design of effective AChE reactivators as antidotes to various organophosphonates requires information on how the reactivators interact with the phosphonylated AChEs. However, such information has not been available hitherto because of three main challenges. First, reactivators are generally flexible in order to change from the ground state to the transition state for reactivation; this flexibility discourages determination of crystal structures of AChE in complex with effective reactivators that are intrinsically disordered. Second, reactivation occurs upon binding of a reactivator to the phosphonylated AChE. Third, the phosphorous conjugate can develop resistance to reactivation. We have identified crystallographic conditions that led to the determination of a crystal structure of the sarin^nonaged^-conjugated mouse AChE in complex with [(*E*)-[1-[(4-carbamoylpyridin-1-ium-1-yl)methoxymethyl]pyridin-2-ylidene]methyl]-oxoazanium dichloride (HI-6) at a resolution of 2.2 Å. In this structure, the carboxyamino-pyridinium ring of HI-6 is sandwiched by Tyr124 and Trp286, however, the oxime-pyridinium ring is disordered. By combining crystallography with microsecond molecular dynamics simulation, we determined the oxime-pyridinium ring structure, which shows that the oxime group of HI-6 can form a hydrogen-bond network to the sarin isopropyl ether oxygen, and a water molecule is able to form a hydrogen bond to the catalytic histidine residue and subsequently deprotonates the oxime for reactivation. These results offer insights into the reactivation mechanism of HI-6 and design of better reactivators.

## Introduction

**Figure 1:**
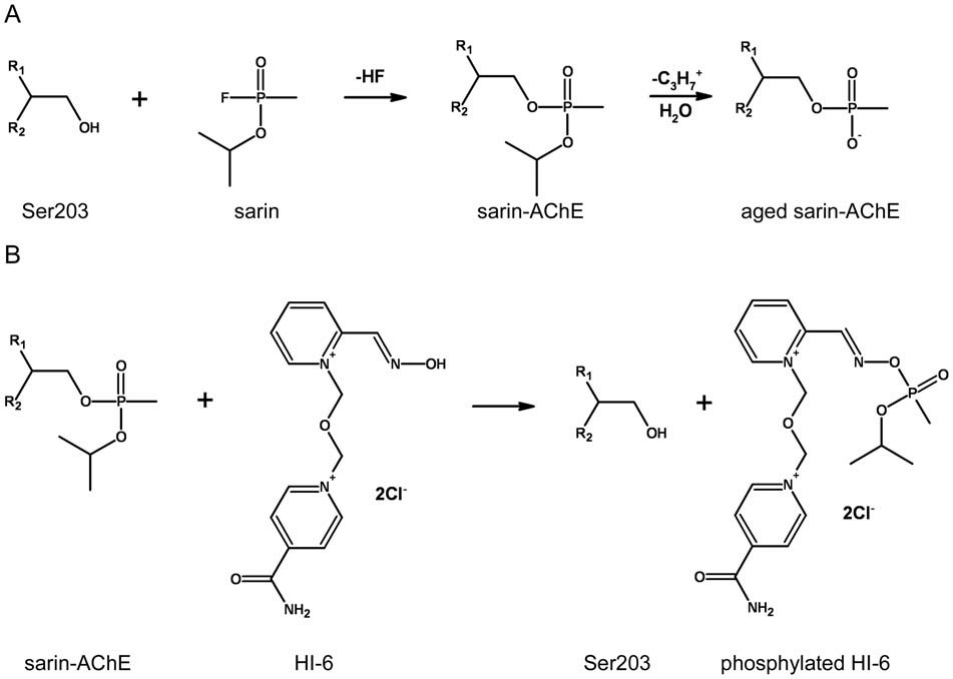
Inhibition and aging of AChE exemplified by the nerve agent sarin (A) and reactivation of sarin-inhibited AChE by HI-6 (B).

Acetylcholinesterase (AChE) terminates cholinergic transmission by rapidly hydrolyzing the neurotransmitter acetylcholine, and it is an essential enzyme for humans and other species [1]. The catalytic triad in this enzyme consists of Ser203, His447 and Glu334 located at the bottom of a ∼20-Å-deep active-site gorge [2], [3], where the residue numbers are based on the sequence numbering of mouse AChE (*m*AChE) that is used throughout this paper. Organophosphates or organophosphonates (OPs), used as insecticides and nerve agents, irreversibly inhibit AChE by phosphonylating the catalytic serine residue (Figure 1) [1]. One key step of current treatment of OP intoxication is to use oximes as nucleophiles to reactivate AChE by removing the phosphorous conjugate [4]. The oxime therapy is, however, limited by two main complications in addition to limitations such as low membrane permeability of oximes containing quaternary ammoniums. First, the reactivation efficiency depends on structures of OPs and reactivators as well as sequence variations in the AChE active site [5]-[10]. For example, [(*E*)-[1-[(4-carbamoylpyridin-1-ium-1-yl)methoxymethyl]pyridin-2-ylidene]methyl]-oxoazanium dichloride (HI-6) is an efficient oxime reactivator of AChEs conjugated to isopropyl metylphosphonofluoridate (sarin) but not to AChEs conjugated to ethyl *N,N*-dimethylphosphoramidocyanidate (tabun) [8]. Reactivator oxo-[[1-[[4-(oxoazaniumylmethylidene)pyridin-1-yl]methoxymethyl]pyridin-4-ylidene]methyl]azanium (obidoxime) reactivates both sarin- and tabun-conjugated AChEs more effectively than HI-6. Second, many OP conjugates can undergo an “aging” process that eliminates one of their substituents (Figure 1). Aged conjugates are resistant to current oxime reactivators [11], [12]. Therefore, more effective AChE reactivators as antidotes to a wide range of OPs are highly desirable.

Oximes such as [(*E*)-(1-methylpyridin-2-ylidene)methyl]-oxoazanium chloride (pralidoxime), HI-6, and obidoxime have been used in the clinic to treat intoxication by certain OPs for decades [13], [14], but little is known about reactivation mechanisms for these oximes at the structural level. For example, the p*K*_a_ values of these oximes are 7.3–8.0 [15]–[17]. These oximes are likely protonated in the AChE active site, although deprotonated oximes in AChE have been proposed [18]. If oximes are protonated in AChE, where is the base that deprotonates the oximes before reactivation? If oximes have relatively low p*K*_a_ values and are deprotonated in the absence of a base in the active site, can these oximes be more nucleophilic than the catalytic serine hydroxyl group to drive the reactivation to completion? Furthermore, there is no available information on how the reactivators change from the Michaelis-Menten state to the transition state for reactivation, wherein the Michaelis-Menten state is defined as the ground state that can directly proceed to the transition state of the chemical reaction [19].

The lack of such information has hampered the structure-based design of more effective AChE reactivators. In our view, this paucity is due to three main challenges. First, unlike enzyme inhibitors, reactivators have to be flexible or mobile in order to be able to change from the Michaelis-Menten state to the transition state; this flexibility often translates to mobility that discourages determination of crystal structures of AChE in complex with effective reactivators because the reactivators are intrinsically disordered. Second, reactivation occurs immediately upon binding of a reactivator to the phosphonylated AChE. Third, aging can occur before reactivation, which enables determination of complexes of reactivator-bound AChEs with aged conjugates but prevents obtaining direct information with regard to reactivation mechanisms.

In this article, we report a structure of the sarin^nonaged^-conjugated *m*AChE in complex with HI-6 (HI-6•sarin^nonaged^-*m*AChE) at the Michaelis-Menten state and supporting crystal structures of the corresponding aged complex (HI-6•sarin^aged^-*m*AChE) and *m*AChE in complex with (*E*)-4-carbamoyl-1-{3-[4-(hydroxyiminomethyl)pyridinium-1-yl]propyl}pyridinium bromide (K027•*m*AChE). We also report our approach of combining crystallography with microsecond molecular dynamics simulation used to determine the Michaelis-Menten complex structure and supportive reactivation kinetics results. We discuss the insights from these structures into the reactivation mechanism of HI-6 and design of improved reactivators.

## Results

### General description of three reactivator-bound *m*AChE crystal structures

The structures of HI-6•sarin^nonaged^-*m*AChE, HI-6•sarin^aged^-*m*AChE, and K027•*m*AChE are generally very similar to that of *apo m*AChE [20]. However, relative to the *apo* structure, a large structural change of Trp286 is seen in HI-6•sarin^nonaged^-*m*AChE and K027•*m*AChE; Trp286 is disordered in HI-6•sarin^aged^-*m*AChE (see below); minor structural changes are also observed for Tyr337 and Tyr341. Similar to other reported *m*AChE structures [20]–[26], the loop region between residues 258–264 is not visible in the electron density map; the loop region of residues 490–498, which is located distant from the catalytic site, is poorly defined. For HI-6•sarin^nonaged^-*m*AChE residues 490–498 were omitted at the end of the refinement. The K027•*m*AChE and HI-6•sarin^aged^-*m*AChE structures contains 5 residues listed in outlier regions of the Ramachandran plot (Table 1). The Protein Data Bank (PDB) entry codes for HI-6•sarin^nonaged^-*m*AChE, HI-6•sarin^aged^-*m*AChE, and K027•*m*AChE are 2WHP, 2WHQ, and 2WHR, respectively.

**Table 1:** Data Collection and Refinement Statistics.

Data collection	HI-6•sarin^nonaged^-*m*AChE	HI-6• sarin^aged^-*m*AChE	K027•*m*AChE	K027PK•*m*AChE
PDB entry code	2WHP	2WHQ	2WHR	–
Wavelength	1.041	1.041	1.131	0.919
Space group	P2_1_2_1_2_1_	P2_1_2_1_2_1_	P2_1_2_1_2_1_	P2_1_2_1_2_1_
Unit cell	79.3×112.2×227.0	79.2×112.3×227.1	79.6×112.3×227.1	77.4×110.7×227.5
Resolution (Å)	29.8–2.2 (2.32–2.2)	29.2–2.2 (2.3–2.15)	19.8–2.6 (2.7–2.54)	29.3–3.2 (3.4–3.2)
Total no. of refl.	765502 (111128)	633429 (91067)	463807 (46633)	239669 (35230)
Unique refl.	102346 (14724)	110895 (16023)	64776 (8351)	33083 (4774)
Completeness	99.2 (98.9)	99.7 (99.9)	98.0 (87.7)	99.9 (100.0)
Multiplicity	7.5 (7.5)	5.7 (5.7)	7.2 (5.6)	3.8 (3.8)
*R*_merge_ 1	0.065 (0.464)	0.065 (0.436)	0.055 (0.290)	0.105 (0.359)
Mean(I)/sd(I)	21.9 (5.1)	15.3 (4.5)	27.1 (7.2)	14.7 (6.2)
Refinement				
*R*-factor^2^/*R*_free_ 3	0.180/0.210	0.190/0.210	0.170/0.220	–
RMS bonds (Å)	0.013	0.010	0.004	–
RMS angles (°)	1.40	1.20	0.90	–
Ramachandran plot^4^%/no. of residues				
Favored	98.1/1022	97.3/1026	96.3/1021	–
Allowed	1.9/20	2.2/23	3.2/34	–
Outlier	0/0	0.5/5^5^	0.5/5^6^	–

1 R*_merge_* = (∑|*I* – <*I*>|)/∑*I*, where *I* is the observed intensity and <*I*> is the average intensity obtained after multiple observations of symmetry related reflections.

2 R-factor = (∑||*F_o_*| – |*F_c_*||)/∑|*F_o_*|, where *F_o_* and *F_c_* are observed and calculated structure factors, respectively.

3 R*_free_* uses 2% randomly chosen reflections defined in Brunger [50].

4 The Ramachandran plots were determined using the program Rampage [56].

5 Residues listed as outliers are A Gly002, B Asp494, B Ser495, B Lys496, and B Ser497.

6 Residues listed as outliers are A Gly342, B Gly342 , A Asp494, B Lys496, and B Ser497.

### Crystal structure of HI-6•sarin^nonaged^-*m*AChE

**Figure 2:**
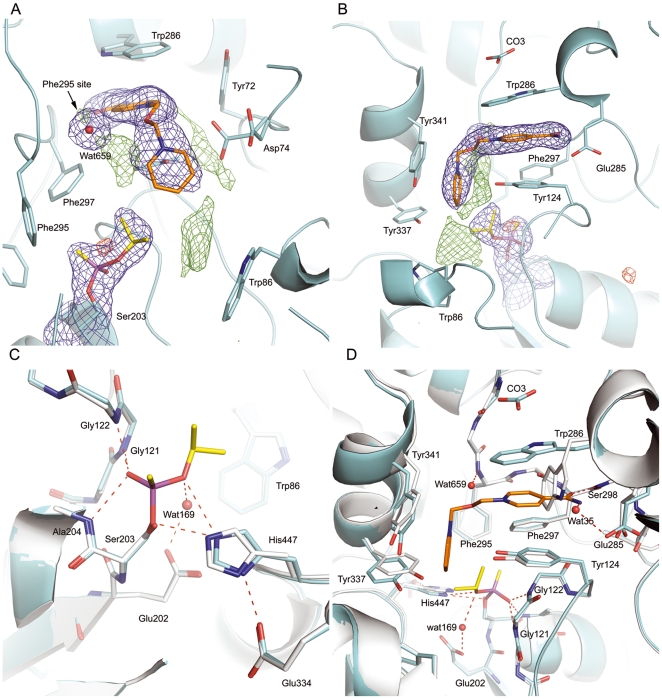
The final electron density map of HI-6•sarin^nonaged^-*m*AChE (A and B) and close-up views for conjugation of sarin^nonaged^ (C) and binding of HI-6 (D) to *m*AChE that is superimposed to the *apo m*AChE. Since the oxime O1, N1 and C1 atoms could not be unambiguously defined, they are not included in the final structure. AChE, sarin, and HI-6 are shown in cyan, yellow and orange, respectively. Putative hydrogen bonding interactions are shown with red dashed lines. In A and B, the 2|*F_o_*| – |*F_c_*| map is contoured at 1σ (blue), and the |*F_o_*| – |*F_c_*| map is contoured at 3.5σ (green) and −3.5σ (red). The *apo m*AChE is shown in grey (C and D). A cross-eyed stereo view of HI-6•sarin^nonaged^-*m*AChE is shown in supporting information Figure S1 and an omit map is shown in supporting information Figure S2.

Seven datasets were collected after pre-treatment of the *m*AChE crystals with sarin for 30 minutes followed by 1-, 2-, 3-, 3-, 4-, 5-, and 10-minute exposures of the sarin^nonaged^-*m*AChE crystals to HI-6, respectively. The structure from the dataset with 1-minute exposure to HI-6 was determined to a resolution of 2.2 Å (Table 1). The structures from the remaining six datasets were refined by 20 cycles of rigid-body refinement followed by 20 cycles of restrained refinement. In the electron density maps from the 2- and 3-minute datasets, sarin is also well defined. However, the structures from datasets with 2–10 minute exposures to HI-6 were not further refined as they are not as good as the 1-minute dataset in terms of the quality of the electron density that defines sarin. In the initial electron density map from the 1-minute dataset, there is a high-density feature corresponding to the phosphorus atom covalently bound to the Ser203 hydroxyl oxygen atom. In the maps from other datasets, the occupancy of the phosphorus atom decreases as the exposure time increases, presumably due to the progress of reactivation. Gratifyingly, the final electron density map from the 1-minute dataset is of good quality and clearly defines the sarin conjugate including its isopropoxy chain (Figures 2A and B).

Similar to a previously determined structure of the sarin^nonaged^-conjugated *m*AChE (sarin^nonaged^-*m*AChE) [26], in HI-6**•**sarin^nonaged^-*m*AChE, the –P = O oxygen atom is hydrogen-bonding to main-chain nitrogen atoms of Gly121 (2.9 Å), Gly122 (2.7 Å) and Ala204 (2.8 Å), namely, the phosphonyl oxygen atom is anchored at the oxyanion hole (Figures 2C and supporting information Figure S1); the methyl group of sarin is accommodated in the acyl pocket surrounded by aromatic rings of Trp236, Phe295, and Phe297; the isopropoxyl group is aligned along the axis of the active-site gorge with its oxygen atom hydrogen-bonding to a water molecule (Wat169, 2.8 Å) and to the N^ε^ atom of His447 (3.1 Å) that forms a catalytic triad with Ser203 (the distance of N^ε^ to O^γ^ is 3.0 Å) and Glu334 (the distance of N^δ^ to O^ε^ is 2.6 Å).

In the HI-6•sarin^nonaged^-*m*AChE structure, the carboxyamino-pyridinium ring of HI-6 is sandwiched by cation-pi interactions from side chains of Tyr124 and Trp286. The side chain of Trp286 undergoes a large conformational change relative to the *apo m*AChE (Figure 2D and supporting information Figures S1 and S2). On the surface of the protein, there is an electron density located near the indole ring of Trp286. Modelling this density feature as a carbonate ion resulted in good correlation to the crystallographic data and this ligand was included in the final model. The carboxyamino oxygen atom of HI-6 forms a hydrogen bond to the main-chain nitrogen atom of Ser298 (2.9 Å), while the carboxyamino nitrogen atom interacts with the side-chain of Glu285 via a water-mediated hydrogen bond network (Wat35, 3.0 Å to the nitrogen atom and 2.7 Å to the carboxyl oxygen atom). The electron density for the side chain of Asp74 is disordered and thus modelled in two conformations: one is similar to that found in the *apo m*AChE structure, and the other, which is dominant, point with its carboxyl oxygen toward the central linker of HI-6. This interpretation resulted in a residual positive density in the vicinity of Asp74 that may account for a low-occupancy position for the oxime moiety of HI-6 (Figure 2A), as shown by the microsecond molecular dynamics simulations of HI-6•sarin^nonaged^-*m*AChE described below. The oxime-pyridinium ring of HI-6 enters the catalytic site and forms cation-pi interactions with side chains of Tyr124, Tyr337, Phe338, and Tyr341. The two carbon atoms of HI-6 that are near Tyr337 are not well defined by the electron density map; these atoms clash with the side chain of Tyr337 (3.1 Å) and have high B factors. These observations suggest that the oxime-pyridinium ring is rather mobile (adopting multiple conformations) in the active-site gorge. In addition, a minor main-chain displacement for Tyr341 was observed, which is presumably caused by unfavourable interactions between the oxime-substituted pyridinium ring and the side chain of Tyr341.

Interestingly, there is a high electron density near the main-chain nitrogen of Phe295 (N^Phe295^). The high-density feature region is hereafter referred to as the Phe295 site. In a previously determined structure of HI-6 in complex with the non-phosphonylated *m*AChE (HI-6•*m*AChE), the oxime oxygen atom was modelled into the same density feature [24]. During the initial refinement of the HI-6•sarin^nonaged^-*m*AChE complex, the oxime group was modelled into the Phe295 site. However, the |*F_o_*| – |*F_c_*| electron density map (contoured at 5σ) showed a residual positive difference electron density at the Phe295 site and poor connectivity between the oxime oxygen atom and the pyridinium ring. On the other hand, modelling a chloride at the Phe295 site and placing the oxime group close to Asp74, showed no residual positive or negative electron density in the Phe295 site, but the oxime moiety shows a peak in the negative difference electron density map and a steric clash between the oxime oxygen atom and the beta carbon atom of Asp74. Furthermore, there is a weak electron density that connects the oxime-pyridinium ring to the side chain of Tyr341 in the B-monomer of the asymmetric unit, possibly indicating that a small population of HI-6 has the oxime moiety pointing toward the aromatic ring of Tyr341, as shown by the microsecond molecular dynamics simulations described below. Subsequent refinement of a model with the oxime moiety pointing toward the side chain of Tyr341 resulted in a steric clash between the oxime and the tyrosine side chain. To investigate whether a halide occupies the Phe295 site, we collected three datasets for the HI-6•BR•*m*AChE complex using a soaking buffer containing 50 mM HI-6 and 1 M KBr. This experiment was inspired by the observation of a well-defined bromide, which is hydrogen bonding to N^Phe295^, in a supporting crystal structure of a non-phosphonylated *m*AChE in complex of K027 (see below). These datasets were collected at wavelengths of 0.91905, 0.91944, and 1.80000 Å, respectively, allowing identification of bromide and chloride [27]. However, analysis of anomalous difference Fourier maps calculated from these datasets did not show any occupancy of either bromide or chloride (data not shown).

Because of no conclusive evidence for the identity of the electron density feature at the Phe295 site, in the final model of the HI-6•sarin^nonaged^-*m*AChE complex, we did not include the oxime moiety and instead modelled a water molecule (Wat659) at the Phe295 site. This interpretation resulted in a residual positive electron density located between the Phe295 site and the oxime-pyridinium ring (Figure 2A). This density feature may represent the oxime moiety of a fraction of HI-6 with its oxime oxygen at a distance of ∼2.9 Å away from N^Phe295^ (data not shown). Residual positive electron densities were also found in the vicinity Asp74 (Figure 2A). As supported by the microsecond molecular dynamics simulations described below, these features may represent minor populations of the oxime moiety. In addition, a low-occupancy HI-6 binding site at the surface of the protein is implied by several residual positive electron densities found close to the His287-Ser293 loop region.

There is a residual positive electron density found at a location that is above the indole ring of Trp86 and close to the nonaged sarin (Figure 2A). An interpretation of this electron density as the binding of HI-6 in the vicinity of Trp86 and the intact sarin is, however, not supported by the distance of ∼3.0 Å between the pyridinium-oxime ring of HI-6 and the sarin *O*-isopropyl chain (see supporting information Figure S3). Such a short distance would cause a severe steric clash. This interpretation is not supported by the simulation described below either. Alternatively, the electron density could suggest that a small fraction of the reactivation product, phosphonylated HI-6, binds in the vicinity of Trp86. However, we did not observe an increase of the residual electron density in subsequent datasets with longer HI-6-exposure times, while we observed that the occupancy of the phosphorus atom decreases as the HI-6 exposure time increases. A satisfactory interpretation of these observations is the binding of HI-6 in a small fraction of an enzyme with an aged sarin as shown in the HI-6•sarin^aged^-*m*AChE crystal structure described below. This interpretation is supported by the observation of a negative electron density (visible at a contour level of –4σ, Figure 2A) close to the phosphorus atom of the conjugate that is likely associated with a small fraction of an aged enzyme. The loss of the isopropyl moiety in a small fraction of the aged enzyme provides space and negative charge to accommodate the cationic pyridinium-oxime ring in the vicinity of Trp86 and the aged sarin, as supported by the superposition of HI-6•sarin^nonaged^-*m*AChE to HI-6•sarin^aged^-*m*AChE (see supporting information S3) The aging half-life for sarin in *m*AChE is presumably close to that of sarin in *h*AChE (∼3 hours) [8], and we found that the half-life of sarin^nonaged^-*m*AChE in the presence of HI-6 is ∼4.6 minutes. These half-life values and the seven datasets with the HI-6-exposure times less than 10 minutes explain why we observed only the change of the electron density for the phosphorus atom in the sarin^nonaged^-*m*AChE in time, adding further support to the interpretation of the residual electron density as the binding of HI-6 in a small fraction of the sarin^aged^-*m*AChE.

### Crystal structure of HI-6•sarin^aged^-*m*AChE

**Figure 3:**
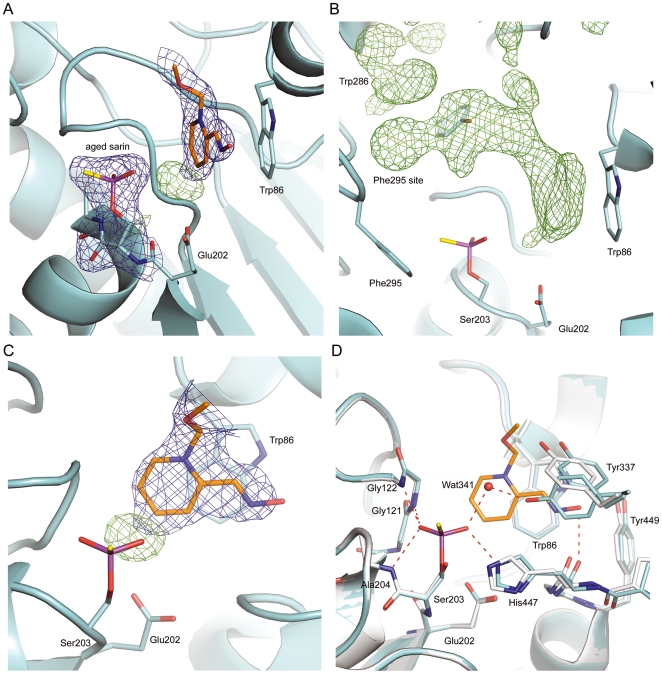
The electron density map of HI-6•sarin^aged^-*m*AChE shown in blue and the positive difference map shown in green (A). The omit map (contour level 3σ) calculated after simulated annealing of a model in which HI-6 and the side chain of Trp286 were omitted (B). The electron density map defining most atoms of the oxime-pyridinium ring of HI-6 (C). Alignment of HI-6•sarin^aged^-*m*AChE (cyan) to the *apo m*AChE (grey) with putative hydrogen bonds shown with red dashed lines (D).

To further support the interpretation of the residual electron density found above the indole ring of Trp86 in HI-6•sarin^nonaged^-*m*AChE, we investigated the binding of HI-6 in the corresponding aged enzyme complex (HI-6•sarin^aged^-*m*AChE) by a crystal structure analysis to a resolution of 2.2 Å (Table 1). The dealkylated form of sarin was explicitly resolved in the 2|*F_o_*| – |*F_c_*| and |*F_o_*| – |*F_c_*| electron density maps (Figure 3A). The –P = O oxygen atom forms hydrogen bonds to the main-chain nitrogen atoms of Gly121 (2.7 Å), Gly122 (2.7 Å), and Ala204 (2.9 Å). The methyl carbon atom is accommodated in the acyl pocket, close to the side chains of Trp236, Phe295 and Phe297. The oxyanion of the aged sarin conjugate forms hydrogen bonds with the N^ε^ atom of His447 (2.7 Å) and with a water molecule (Wat341, 2.9 Å).

A visible electron density feature spans the active-site gorge of *m*AChE (Figure 3B). In contrast to the HI-6•sarin^nonaged^-*m*AChE crystal structure, the oxime-pyridinium ring of HI-6 in the aged enzyme is ordered whereas the carboxyamino-pyridinium portion is disordered. The oxime-pyridinium ring is engaged in a parallel, cation-pi interaction with the indole ring of Trp86 (Figure 3) and a hydrogen bond between the oxime oxygen atom and the main-chain oxygen atom of His447 (3.2 Å). A positive electron density feature, most likely a low-occupancy water molecule (corresponding to Wat752 in the *apo m*AChE crystal structure), was found close to the oxime-pyridinium ring (Figures 3A and 3C). The electron density map of Tyr337 is slightly disordered, suggesting that Tyr337 adopts two side-chain conformations. One is similar to the conformation found in the *apo m*AChE crystal structure and it is termed *apo* conformation. The other has the side chain pushed toward His447 by the oxime-pyridinium ring of HI-6. There is a steric clash between the oxime-pyridinium ring and the side-chain of Tyr337 in its *apo* conformation. There is an additional positive electron density close to the phenolic oxygen atom of Tyr337 in its *apo* conformation, which may account for a counter ion or an alternative, low-occupancy HI-6 conformation with the oxime-pyridinium ring parallel to Trp86. The remaining active-site gorge contains 2|*F_o_*| – |*F_c_*| and |*F_o_*| – |*F_c_*| electron density features that could not be unambiguously modelled (Figure 3B).

The final model of HI-6•sarin^aged^-*m*AChE contains well-defined atoms, namely, the aged sarin and the HI-6 oxime-pyridinium parallel to Trp86. The carboxyamino-pyridinium ring of HI-6 bound above the indole ring of Trp86, HI-6 molecules bound alternatively, and the side chain of Trp286 were omitted from the final model. The occupancy of the oxime-pyridinium ring is set to 0.7 and the occupancies of the *apo*-like and HI-6-accommodating side-chain conformations of Tyr337 are set to 0.3 and 0.7, respectively in the final model.

### Crystal structure of K027•*m*AChE

To investigate the identity of the electron density feature at the Phe295 site in HI-6•sarin^nonaged^-*m*AChE, we determined the 2.6-Å crystal structure of a non-phosphonylated *m*AChE in complex with K027, a close analogue of HI-6 that is less effective in reactivating the sarin^nonaged^-*h*AChE than HI-6 (Tables 1 and 3). The key structural difference between HI-6 and K027 is that the oxime moiety is substituted at the 2-position of the pyridinium ring of HI-6 and at the 4-position for K027. The *para* substitution prevents the presence of the oxime group at the Phe295 site when K027 binds at the *m*AChE active site in a similar fashion as HI-6.

**Figure 4:**
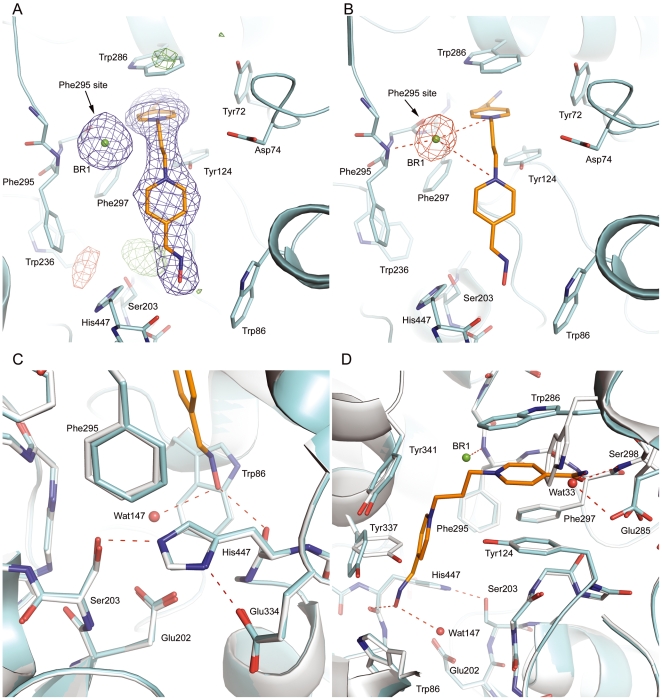
The final 2|*F_o_*| – |*F_c_*| electron density map of K027•*m*AChE shown in blue and the positive difference map shown in green (A). The anomalous difference Fourier map (red) clearly indicating that a bromide ion occupies the Phe295N site (B). The binding of K027 (orange) within the active-site gorge of *m*AChE (C and D). The oxygen and nitrogen atoms are shown in red and blue, respectively. Water molecules are shown with red spheres. K027•*m*AChE and the *apo m*AChE are shown in cyan and grey, respectively. The 2|*F_o_*| – |*F_c_*|, |*F_o_*| – |*F_c_*|, and anomalous difference Fourier maps are contoured at 1σ,±3.5σ, and 10σ, respectively.

Indeed, similar to the binding of HI-6 in sarin^nonaged^-*m*AChE (Figure 4), in the K027•*m*AChE complex, the carboxyamino-pyridinium ring of K027 is sandwiched by Tyr124 and Trp286, and the 4-oxime-pyridinium ring enters the catalytic site where it is found parallel to Tyr337 with its oxime oxygen forming a 2.9-Å hydrogen bond to the main-chain oxygen of His447 and a 3.1-Å hydrogen bond to a water molecule (Wat147). Interestingly, in contrast to HI-6•sarin^nonaged^-*m*AChE, Asp74 adopts the side-chain conformation found in the *apo m*AChE and two low-occupancy water molecules (Wat626 and Wat628) seem to bridge the interaction between K027 and Asp74.

There is also a high-density feature at the Phe295 site in the K027•*m*AChE complex. Because K027 was added to the crystals in the K027•HBr salt form, and because the 4-substituted oxime group cannot access the Phe295 site, the spherical feature was initially modelled as a bromide, resulting in good correlation to the crystallographic data. This interpretation is further supported by the anomalous difference Fourier map calculated from dataset K027PK•*m*AChE (Table 1) collected at a wavelength of 0.91944 Å, close to the bromide *K*-edge. The anomalous difference Fourier map shows a strong signal, visible at a contour level of 18σ, at the Phe295 site (Figure 4B). Thus, in the final model of the K027•*m*AChE complex, the Phe295 site is occupied by a bromide ion (BR1) with a distance of 3.3 Å to N^Phe295^ and 3.2 Å to a water molecule (Wat541). The distance of BR1 to the nitrogen atoms of the 4-oxime- and 4-carboxyamino-substituted pyridinium rings of K027 are 4.6 and 4.1 Å, respectively (Figure 4). A positive residual electron density suggests that an unidentified ion interacts with the hydroxyl oxygen of the catalytic residue Ser203.

### Apparent p*K*_a_ of HI-6

The pK_a_ value of HI-6 was reported to be 7.28 [16], which is chemically counterintuitive because the p*K*_a_ values of close analogues pralidoxime, obidoxime, and 1,n-alkylene-bis-*N*,*N*'-2-pyridiniumaldoxime (n = 3–6) are 7.8–8.0 [15], [17], [28]. We accordingly measured the apparent p*K*_a_ value of HI-6 using proton nuclear magnetic resonance (NMR) spectra and found that the p*K*_a_ value of HI-6 is 7.63 (supporting information Figure S4).

### Microsecond molecular dynamics simulation of HI-6•sarin^nonaged^-*m*AChE

Inspired by a number of reported molecular dynamics simulations [29]–[32], we performed 100 10-ns-long molecular dynamics simulations (each with 1.0-fs time step and different initial velocities) using an HI-6•sarin^nonaged^-*m*AChE crystal structure, which was partially refined from the dataset with 1-minute exposure to HI-6, as a starting structure. This study was carried out to determine popular conformations of the oxime-pyridinium ring of HI-6 that is disordered in the crystal structure. In the starting structure, the oxime oxygen atom was protonated (in the neutral form) according to its p*K*_a_ of 7.63 and purposely placed 3.7 Å and 7.2 Å away from the phosphorus atom and from N^Phe295^, respectively, by rotating two torsions along -N-CH2-O-. Otherwise, the starting structure was the same as the partially refined crystal structure. There was no water molecule placed to form a hydrogen bond to N^Phe295^ in the starting structure. The reason to perform 100 10-ns-long simulations instead of one 1.0-ms-long simulation was twofold. First, we found that the sampling of multiple short simulations is more efficient than the sampling of a single long simulation using the same simulation protocol described in the Method section. Second, we also found that a large conformational change occurred to the Trp86-containing helix after 50 ns of our simulations of HI-6•sarin^nonaged^-*m*AChE, which could relate to the reported back door opening of the AChE active site [33], [34] or indicate an artefact of the simulation protocol that has not been evaluated rigorously for simulations longer than 20 ns.

A total of 2,000 instantaneous structures collected at 50-ps intervals during the last 1.0-ns period of the 100 simulations were subjected to a first-round cluster analysis using the averagelinkage algorithm (epsilon = 2.0 Å and RMS on alpha-carbon atoms) [35]. The instantaneous structures and an average of the instantaneous structures are hereafter termed conformers and an average conformer, respectively. This analysis identified six clusters of conformers. The number of conformers in Clusters 1–6 is 40, 60, 1800, 60, 20, and 20, respectively. Visual inspection of the average conformer of the third cluster revealed a very distorted loop containing residues 74–78 implying the loop is highly mobile. This loop is connected to a helix that is directly involved in crystal packing. This loop is also in contact with HI-6 in the crystal structure.

**Figure 5:**
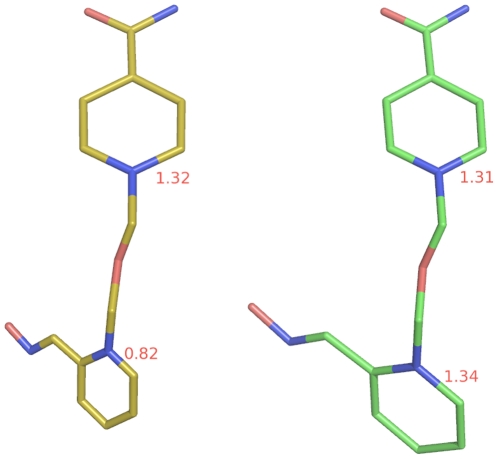
The HI-6 conformations in A2C (yellow) and A3C (green) showing the disordered and ordered oxime-pyridinium rings in A2C and A3C, respectively.

All 1800 conformers from the most populated cluster (Cluster 3) of the first-round cluster analysis were subsequently subjected to a second-round cluster analysis using the averagelinkage algorithm (epsilon = 1.5 Å and RMS on all atoms of residues 74–78). This analysis identified 49 clusters. The number of conformers in Clusters 1–5 is 400, 335, 89, 188, and 146, respectively, while the numbers of conformers in the remaining clusters are all less than 89. Interestingly, the average conformer (denoted as A2C, see supporting information Dataset S1) of the most populated cluster (Cluster 1) of the second-round cluster analysis is similar to the final model of HI-6•sarin^nonaged^-*m*AChE including the loop containing residues 74–78; the root mean square deviation (RMSD) of all alpha-carbon atoms between the two is 1.24 Å. It is worth noting that no energy minimization was performed on A2C. This large RMSD difference is, however, caused partly by crystal packing [36], [37] and partly by the uncertainties of residues with high B factors. To minimize the difference caused by crystal packing and high B factors, we propose to use the RMSD of alpha-carbon atoms for residues that are not involved in crystal contact and have B factors of alpha-carbon atoms lower than the average alpha-carbon B factor. This proposed RMSD is hereafter termed cool-and-free RMSD (C&F RMSD). Indeed, the C&F RMSD between the final crystal structure and A2C is 0.56 Å, suggesting that A2C can represent the final model of the crystal structure in solution. In A2C, the oxime-pyridinium ring of HI-6 is contracted by ∼38%, as shown in Figure 5, while the carboxyamino-pyridinium ring is not contracted at all, implying the oxime-pyridinium portion is highly mobile or intrinsically disordered while the carboxyamino-pyridinium portion is stationary or intrinsically ordered. The HI-6 structure with a contracted oxime-pyridinium ring and an uncontracted carboxyamino-pyridinium ring is also observed in the average of all 100,000 conformers collected at 1-ps intervals during the last 1.0-ns period of the 100 simulations. These observations support the above-described interpretations of the electron density map of HI-6•sarin^nonaged^-*m*AChE.

**Figure 6:**
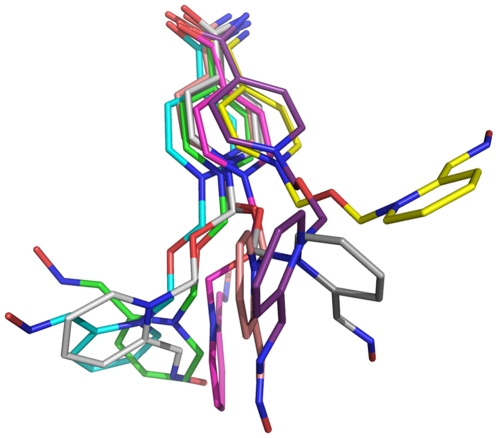
The HI-6 conformations in the average conformers of all 8 clusters from the third-round cluster analysis (green: cluster 1; cyan: cluster 2).

To determine popular conformations of the oxime-pyridinium ring, all 400 conformers from the most populated cluster (Cluster 1) of the second-round cluster analysis were subjected to a third-round cluster analysis using the averagelinkage algorithm (epsilon = 1.0 Å and RMS on all atoms of HI-6). This analysis identified 8 clusters. The number of conformers in Clusters 1–8 is 235, 81, 20, 20, 20, 2, 2, and 20, respectively. As shown in Figure 6, no contraction of the oxime-pyridinium ring was observed in any average conformer of the 8 clusters, noting that no energy minimization was performed on these average conformers. The populations of the 8 clusters and the corresponding HI-6 conformers explain why the oxime-pyridinium ring is contracted but the carboxyamino-pyridinium ring is not in A2C. Furthermore, some HI-6 conformations with the oxime group pointing to Asp74 and Tyr341 are consistent with the afore-mentioned interpretation of the HI-6•sarin^nonaged^-*m*AChE crystal structure. In contrast to the HI-6 conformation in the previously reported HI-6•*m*AChE crystal structure [24], none of the 8 average conformers of HI-6 shown in Figure 6 has the oxime oxygen atom hydrogen bonding to N^Phe295^.

Interestingly, the average conformer (denoted as A3C, see supporting information Dataset S2) of the most populated cluster (Cluster 1) of the third-round cluster analysis has an RMSD of 1.26 Å relative to all alpha-carbon atoms of the final model of the HI-6•sarin^nonaged^-*m*AChE crystal structure. The C&F RMSD between the two structures is 0.55 Å, indicating that A3C is the major composite conformer of A2C, or a major composite conformer of the crystal structure in solution. In A3C, the oxime-pyridinium portion is stationary as it is uncontracted (see Figure 5); the oxime oxygen atom is 6.0 and 6.9 Å away from N^Phe295^ and the phosphorus atom, respectively, although the corresponding distances are 7.3 and 3.7 Å in the starting structure of the simulations.

**Figure 7:**
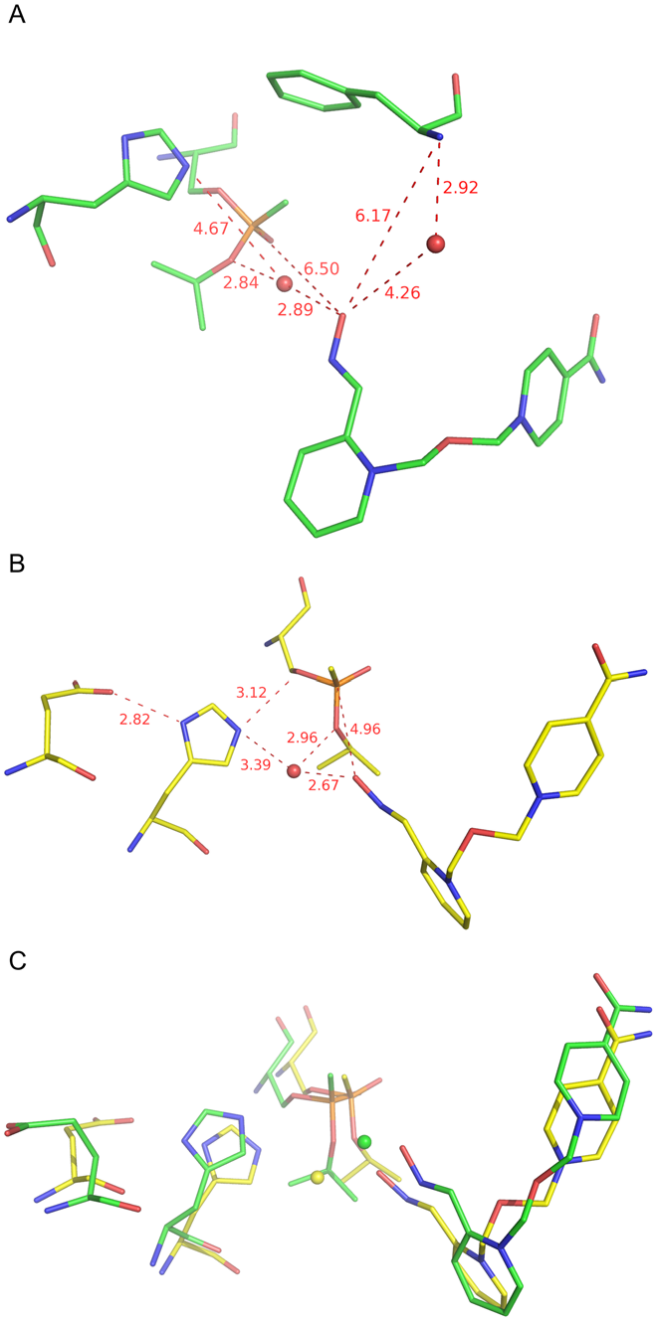
Close-up view of I3Cr (A) and I3C (B) and the overlay of I3Cr and I3C (C). The phenylalanine residue in A is Phe295, the glutamate residue in B and C is Glu334, and the histidine residue in A-C is His447.

To investigate whether there is a possible water molecule that forms a hydrogen bond to N^Phe295^ as suspected the early stage of structural refinement, we visually inspected an instantaneous conformer (denoted as I3Cr, see Figure 7A and supporting information Dataset S3) that was identified to be the closest to A3C by the third-round cluster analysis. Because water molecules are highly contracted in the average conformer A3C, structural information on water has to be obtained from instantaneous structures. I3Cr has a C&F RMSD of 0.51 Å relative to A3C and it is therefore a major composite conformer of A3C. As shown in Figure 7A, in I3Cr, the oxime oxygen atom is 6.2 and 6.5 Å away from N^Phe295^ and the phosphorus atom, respectively; there is indeed a water molecule hydrogen bonding to N^Phe295^, but this water molecule does not form a hydrogen bond or a hydrogen-bond network to the oxime oxygen atom. Instead, the oxime oxygen atom forms a water-mediated hydrogen-bond network to the isopropoxyl oxygen atom. The water molecule is 4.7 Å away from the N^ε^ atom of His447. In contrast to the *apo m*AChE structure, His447 in I3Cr does not have a hydrogen bond to Glu334 that is presumably pushed away by sarin and HI-6 (see Figure 7C).

A distance analysis of the 1800 conformers from the most populated cluster (Cluster 3) of the first-round cluster analysis identified 14 (1%) instantaneous conformers with a distance of the oxime oxygen atom to the phosphorus atom ranging from 5.0 to 5.5 Å. These conformers are similar to I3Cr and they are composite conformers of A3C. These instantaneous conformers reveal the trajectories of the oxime group moving toward the phosphorus atom for reactivation. In one of these, which is denoted as I3C (see Figure 7B and supporting information Dataset S4) and has the shortest distance of 5.0 Å between the oxime oxygen atom and the phosphorus atom, the oxime oxygen atom has a hydrogen-bond network to the N^ε^ atom of His447, the bridging water molecule has a hydrogen bond to the sarin isopropoxyl oxygen atom, His447 has a hydrogen bond to Glu334, the N^ε^ atom is 3.1 Å away from the O^γ^ atom of Ser203, but there is no hydrogen bond between N^ε^ and O^γ^ as both atoms are deprotonated (see Figure 7B). Because I3C has a C&F RMSD of 0.90 Å to the HI-6•sarin^nonaged^-*m*AChE crystal structure, this instantaneous composite conformer is relevant to the HI-6•sarin^nonaged^-*m*AChE crystal structure and sheds light on the reactivation mechanism by HI-6 as will be discussed below.

### Cross-checking the simulation using the diffraction data

**Figure 8:**
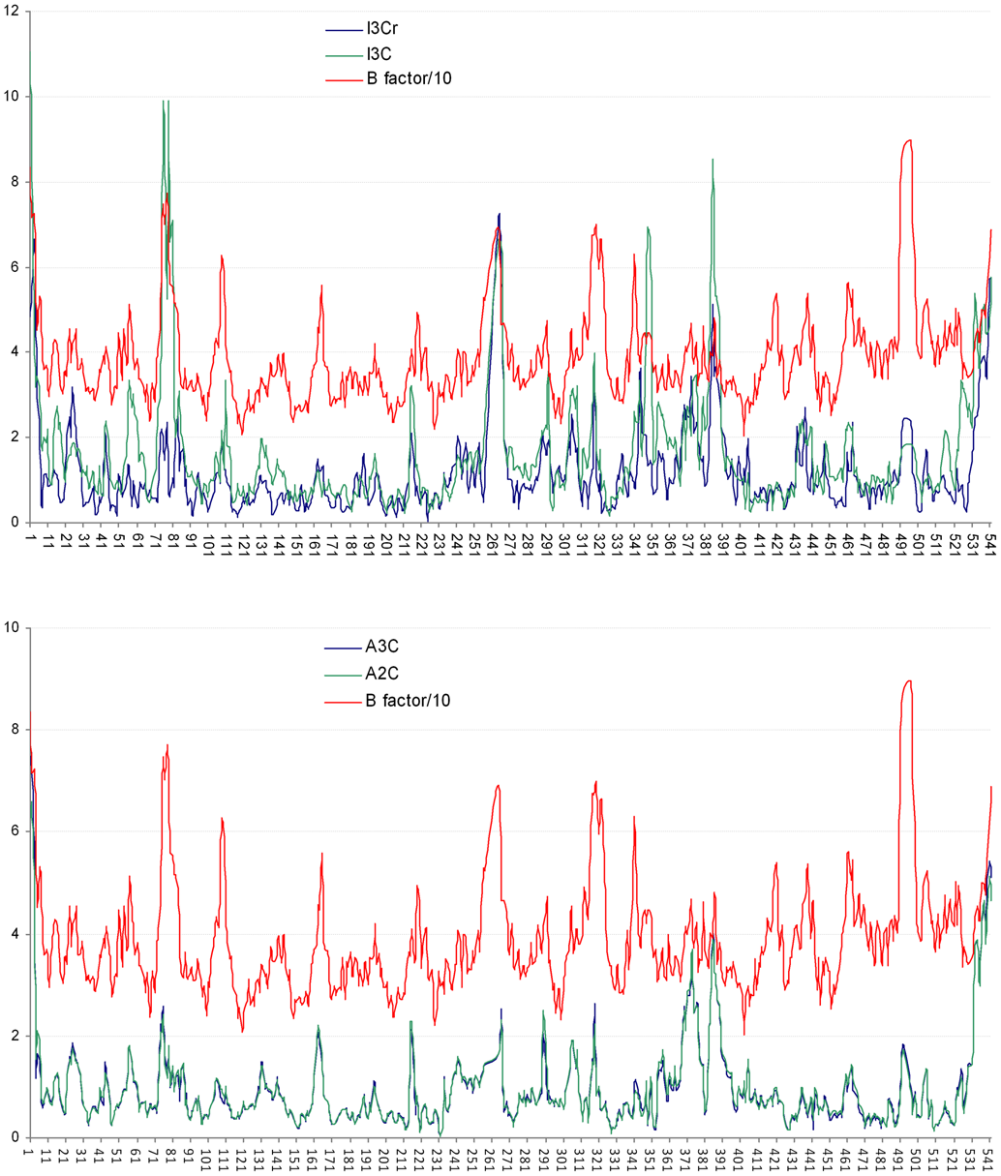
Deviations for alpha-carbon atoms between the HI-6•sarin^nonaged^-*m*AChE crystal structure and the aligned structure of I3Cr (top panel), I3C (top panel), A3C (bottom panel), or A2C (bottom panel), and scaled B factors for the corresponding alpha-carbon atoms of the crystal structure. Horizontal axis: residue ID; vertical axis: alpha-carbon deviation in Å and B factors scaled by 10% in Å^2^.

To cross check the microsecond molecular dynamics simulation of HI-6•sarin^nonaged^-*m*AChE, deviations for all alpha-carbon atoms between an aligned conformer and the final model of the HI-6•sarin^nonaged^-*m*AChE crystal structure (monomer A of the asymmetric unit) were computed and plotted against B factors of their corresponding alpha-carbon atoms (see Figure 8), wherein only alpha-carbon atoms were used for aligning the two structures. As apparent from Figure 8A, the alpha-carbon deviations of average conformers A3C and A2C correlate well with B factors for all residues except for residues 350–356 and 372–385 that are in fact involved in crystal contact. The alpha-carbon deviations of instantaneous conformers I3Cr and I3C also correlate reasonably with B factors (Figure 8B). These correlations are confirmed by C&F RMSDs of average conformers A3C (0.55 Å) and A2C (0.56 Å) relative to the corresponding crystal structure and by the corresponding C&F RMSDs of instantaneous conformers I3Cr (0.67 Å) and I3C (0.90 Å). A3C, A2C, I3Cr, and I3C are therefore in accordance with the experimentally obtained diffraction data.

### Biochemical characterization of HI-6 and K027

To obtain further support for the crystal structures described above, we performed biochemical characterization of HI-6 and K027 using *m*AChE and its mutants. Under the conditions used in the soaking experiments (32% PEG 750 MME, 100 mM HEPES, pH 7.0, 25 mM NaCl at 25°C), we found that the reaction rate constant (*k_r_*) of HI-6 in reactivating the sarin^nonaged^-*m*AChE is ∼0.15 min^−1^ (t_½_ of ∼4.6 min), and the dissociation constant (*K_D_*) of HI-6 for the sarin^nonaged^-*m*AChE is ∼100 µM. Analysis using a partial mixed inhibition scheme showed that HI-6 has *K_i_* values of 32 µM, 20 µM, and 255 µM for the wild-type non-phosphonylated *m*AChE and its Asp74Glu and Tyr341Ala mutants, respectively (Tables 2 and supporting information Figure S5). The Asp74Glu substitution resulted in a 1.6-fold increase in affinity whereas the Tyr341Ala substitution decreased the affinity by 8.2 fold. Under assay conditions similar to the reported ones [8], the *K_D_* and *k_r_* values of K027 are 92 µM and 0.054 min^−1^ for the sarin^nonaged^-*h*AChE and 349 µM and 0.047 min^−1^ for the DFP-*h*AChE, respectively (Tables 3 and supporting information Figure S6). The 3.8-fold decrease in the affinity of K027 for the DFP-*h*AChE compared to sarin^nonaged^-*h*AChE suggests that the binding of K027 involves the Phe295 site, because the reported crystallographic studies showed that the DFP conjugation induces a structural change of the Phe295-containing acyl loop (residues 287–299), whereas the sarin conjugation does not change the acyl loop conformation found in the *apo* AChE [5], [26], [38].

**Table 2:** Analysis of the Binding Constants of Acetylthiocholine and HI-6 to AChE Using a Partial Mixed Inhibition Scheme^3^.

Construct	*K* 1 *_m_* (µM)	*K_i_* 2 (µM)	α	β
*h*AChE	124**±**4	35**±**3	1.2**±**0.1	0.2**±**0.0
*m*AChE	81**±**6	31**±**2	1.9**±**0.2	0.1**±**0.0
Asp74Glu *m*AChE	182**±**6	20**±**2	3.6**±**0.6	0.3**±**0.0
Tyr341Ala *m*AChE	119**±**6	255**±**28	18**±**10	0.8**±**0.2

1 Mean value of 8 independent determinations±S.D.

2 Mean value of at least 4 independent determinations±S.D.

3 See supporting information Figure S5.

## Discussion

### Support from known reactivation studies of AChE mutants

The afore-described HI-6•sarin^nonaged^-*m*AChE structure determined by X-ray crystallography and multiple molecular dynamics simulations (MMDSs) is consistent with reported reactivation studies using HI-6 and conjugated AChEs that are comparable to sarin^nonaged^-*m*AChE [39]–[41]. For example, isopropyl-methyl-phosphonothiocholine (iPrMP) reacts with AChE to yield the same conjugate as sarin. Consistently, a Tyr124Glu mutation of iPrMP*-m*AChE resulted in a 36-fold decrease of the bimolecular reactivation rate constant for the reactivation by HI-6, and a 3-fold reduction was also observed for the Trp286Ala substitution [40]. The involvement of Tyr124 and Trp286 in the HI-6 reactivation is in agreement with our crystal structure and simulations of HI-6•sarin^nonaged^-*m*AChE showing that the two residues sandwiches the carboxyamino-pyridinium ring of HI-6 firmly through cation-pi interactions. In addition, the Tyr337Ala mutant of *m*AChE reduces the bimolecular rate constant by 5.5-fold and displays a 30-fold decrease in affinity [41], which is also consistent with the hydrophobic interaction between Tyr337 and HI-6 shown in the HI-6•sarin^nonaged^-*m*AChE crystal structure and its simulated conformations. Other reported mutagenesis studies suggest that the side chains of Phe295, Phe297, and Trp86 are less critical for the reactivation of iPrMP*-m*AChE by HI-6 [39]–[41] as substitutions of Phe295Leu, Phe297Ile, and Trp86Ala change the bimolecular rate constant by 0.9- to 1.4-fold [40]. Again, the HI-6•sarin^nonaged^-*m*AChE crystal structure (Figure 2A) and its simulations (Figure 6) show no strong interactions between HI-6 and the side chains of these residues. The agreement between the HI-6•sarin^nonaged^-*m*AChE structure and the mutagenesis studies using either iPrMP or sarin described above offers a foundation for the following discussion with regard to the enzymatic state.

### The HI-6•sarin^nonaged^-*m*AChE complex at the Michaelis-Menten state

In this article, we define the ground state that can directly proceed to the transition state of the reactivation reaction as the Michaelis-Menten state. An HI-6-bound *m*AChE conjugated to an aged sarin or a non-phosphonylated *m*AChE in complex with HI-6 is not a complex at the Michaelis-Menten state (hereafter termed Michaelis-Menten complex), as it cannot proceed to the transition state. Unlike enzyme inhibitor design, one of the challenges of enzyme reactivator design is the entrapment of a high-affinity reactivator at the Michaelis-Menten state. While Michaelis-Menten complexes can offer crucial information on how to avoid the entrapment at the Michaelis-Menten state [19], [42], [43], such complexes were previously thought difficult to obtain from crystallographic studies. As reported recently [25] and discussed below, some Michaelis-Menten complexes are obtainable now if using crystallographic approaches that are complemented by MMDSs or other means.

The reactivation of sarin^nonaged^-*m*AChE by HI-6 is a relatively efficient reaction with a rate constant of ∼0.15 min^−1^ (t_½_ of ∼4.6 minutes) under the buffer condition that was used in the crystallographic experiments. This discourages crystallographic determination of the Michaelis-Menten complex because of two competing requirements. First, the inevitable reactivation/aging can significantly decrease the occupancy of the nonaged conjugate if the HI-6-exposure time is around or longer than the half-lives of sarin in the presence and absence of HI-6. Second, a short exposure to the reactivator will limit the diffusion of the reactivator into the enzyme and subsequently reduce the occupancy of the reactivator. Accordingly, we used an HI-6 concentration that is at least 100-fold higher than its *K_D_* (∼100 µM) for HI-6•sarin^nonaged^-*m*AChE to avoid the diffusion problem, and ran a time-course experiment to find the shortest HI-6-exposure time that can minimize the unwanted reactivation and aging and still yield a good-quality electron density map. Knowing the aging half-life of sarin of ∼3 hours for *h*AChE [8] and the half-life of ∼4.6 minutes for sarin in the presence of HI-6, we found that 30-minute exposure to 2 mM of sarin followed by one-minute exposure to HI-6 led to a good-quality electron density map that unequivocally defines a nonaged sarin and most of the HI-6 structure. We also found that longer HI-6-exposure times successively decrease the occupancy of the conjugate, whereas the occupancy of HI-6 remains unaffected (data not shown). The high concentration of HI-6 in the soaking experiment could in theory lead to multiple binding modes (*i.e*., HI-6 occupies low- and high-affinity sites). In this study, we found that the high HI-6 concentration does not interfere with the crystallographic determination of the HI-6•sarin^nonaged^-*m*AChE structure, given the excellent correlation of the crystallographic results to the microsecond molecular dynamics simulation and the biochemical characterizations.

It was more challenging to determine the crystal structure of the sarin^nonaged^-*m*AChE in complex with HI-6 half of which is intrinsically disordered, comparing to the Michaelis-Menten complex of tabun-*m*AChE in complex with 1,7-heptylene-bis-*N*,*N*′-2-pyridiniumaldoxime dichloride (ortho-7•tabun-*m*AChE) [25]. Without knowing the oxime-pyridinium portion of HI-6 is highly mobile in the sarin^nonaged^-*m*AChE active site, our initial and exhaustive attempts to model the oxime moiety in the electron density map were fruitless. The main problem of these attempts was the interpretation of the density feature at the Phe295 site. On the one hand, the following observations suggest that the oxime oxygen atom occupies the Phe295 site with its oxime oxygen atom hydrogen bonding to N^Phe295^: (1) modelling the oxime group away from the Phe295 site and close to Asp74 resulted in a negative peak in the |*F_o_*| – |*F_c_*| map, a steric clash with the beta-carbon atom of Asp74, and no 2|*F_o_*| – |*F_c_*| electron density of the oxime moiety; (2) the Asp74Glu mutation moderately enhanced the affinity of HI-6 for *m*AChE; (3) modelling the oxime group away from the Phe295 site leaning toward Tyr341 resulted in a steric clash with the side chain of Tyr341, but the side chain of Tyr341 shows no obvious signs of large-scale compensatory structural changes; (4) the Tyr341Ala mutation decreased the affinity of HI-6 for the enzyme; (5) reactivation kinetics data of sarin- and DFP-inhibited AChE indicate that HI-6 is more sensitive to the conformational change of the Phe295 site than K027 (Table 3). On the other hand, the initial modelling of the oxime group at the Phe295 site resulted in residual positive difference electron density at the Phe295 site and poor connectivity between the oxime oxygen atom and the pyridinium ring. The disagreement was later reconciled by our microsecond-scale MMDSs of HI-6•sarin^nonaged^-*m*AChE that revealed the following structural and dynamic details.

**Table 3:** Reversible Inhibition (*K_i_*), Dissociation (*K_D_*), and Reaction Rate Constants (*k_r_*). See supporting information Figure S6 for details.

Oxime	*K_i_* (µM)^a^ *h*AChE	*K_D_* (µM) *h*AChE			*k_r_* (min^−1^) *h*AChE		
		^Sarin^	^DFP^	^Ratio^	^Sarin^	^DFP^	^Ratio^
HI-6	35	50.1^b^	1935^b^	1.4/38.6	0.677^b^	0.02^b^	33.9
Obidoxime	–	31.3^b^	63.8^b^	2.0	0.937^b^	0.06^b^	15.6
K027	–	92±22^c^	349±47^c^	3.8	0.054±0.011^c^	0.047±0.003^c^	1.1

a Determined in this study (Table 2).

b Data from [8].

c Determined in this study, mean value±S.D. of 2–4 independent determinations.

First, the MMDSs did not show that the oxime group occupies the Phe295 site with its oxime oxygen atom hydrogen bonding to N^Phe295^. A distance analysis of all 2,000 conformers collected at 50-ps intervals during the last 1.0-ns period of the 100 simulations identified 60 conformers with a hydrogen bond between the oxime oxygen atom and N^Phe295^. This result precludes the concern that the microsecond-scale simulation is perhaps still not long enough to capture the conformations with the hydrogen bond to N^Phe295^. However, according to visual inspection, the main-chain conformations of these unpopular conformers are markedly different from that of the HI-6•sarin^nonaged^-*m*AChE crystal structure. Instead of hydrogen bonding to the oxime oxygen atom, N^Phe295^ has a hydrogen bond to a water molecule in both I3C and I3Cr derived from the MMDSs, although there is no water molecule that forms a hydrogen bond to N^Phe295^ in the starting structure of the simulation. The simulation result is consistent with other AChE crystal structures that reveal a hydrogen bond between a water molecule and N^Phe295^ [20], [25], [44], it is also consistent with re-inspection of our previously reported electron density map of obidoxime-*m*AChE that identified a well-defined water molecule at the Phe295 site [24]. Taken together, the MMDSs do not support the interpretation of the observed electron density map of HI-6•sarin^nonaged^-*m*AChE by modelling of the oxime group of HI-6 at the Phe295 site that resulted in a poor connectivity between the oxime oxygen atom and the pyridinium ring.

Second, the MMDSs showed that the oxime-pyridinium portion of HI-6 is highly mobile and HI-6 adopts 8 major conformations in the sarin^nonaged^-*m*AChE active site (Figure 6). Some of the 8 conformations have the oxime group pointing to Asp74 and Tyr341, whereas the most populated conformation has the oxime group away from Asp74 and Tyr341. The former explains why there is a weak electron density feature that connects the oxime-pyridinium ring to the side chain of Asp74 or Tyr341; the latter explains the above-described observations 1–5 that initially led to the speculation that the oxime group occupies the Phe295 site.

Third, as to average conformers of A2C and A3C and instantaneous conformers I3Cr and I3C derived from the MMDSs, the C&F RMSDs relative to the crystal structure of HI-6•sarin^nonaged^-*m*AChE are 0.56 Å, 0.55 Å, 0.67 Å, and 0.90 Å, respectively. These results indicate that all four conformers have the integrity of the crystal structure.

The 1-minute dataset likely contains structural information more complex than the information we obtained through crystallographic analysis and MMDSs using simplified structural models. For example, as described above, the 1-minute crystal contains a small fraction of the aged sarin conjugate in addition to the dominating nonaged conjugate. Nonetheless, the consistency among the diffraction data, the reactivation kinetics data, and the MMDS results suggests that the HI-6•sarin^nonaged^-*m*AChE crystal structure and the average conformer A3C represent the main structure in the 1-minute crystal and in solution, respectively. It also suggests that the HI-6•sarin^nonaged^-*m*AChE crystal structure supplemented with I3C is a plausible Michaelis-Menten complex that offers insights into reactivation mechanism and reactivator design as discussed below.

### Insight into the reactivation mechanism of HI-6

**Figure 9:**
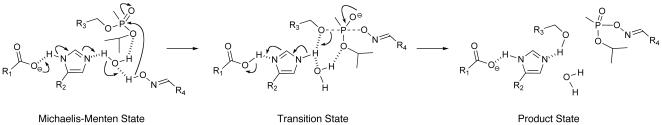
Proposed reactivation mechanism of sarin^nonaged^-*m*AChE by HI-6. Dashed lines represent the bond between the phosphorous atom and the attacking or leaving atom. Hashed lines show hydrogen bonds.

Given the HI-6•sarin^nonaged^-*m*AChE crystal structure and the I3C model, a reactivation mechanism of HI-6 can now be proposed at the atomic level (Figure 9). In I3C at the Michaelis-Menten state (Figure 7B), the oxime oxygen atom has a hydrogen-bond network to His447, the bridging water molecule and His447 have hydrogen bonds to the sarin isopropyl ether oxygen atom and Glu334, respectively. This structural arrangement positions the oxime oxygen atom 5.0 Å away from the sarin phosphorus atom before reaching the transition state (Figure 7B). There is a hydrogen-bond network of Glu334-His447-Wat-Oxime (termed the catalytic tetrad, Figure 9), which is akin to the hydrogen-bond network of the catalytic triad (Glu334-His447-Ser203). In the same way as the triad deprotonates the hydroxyl group of Ser203, the tetrad deprotonates the oxime hydroxyl group and makes the oxime a strong nucleophile for attacking the phosphorus atom that is 5.0 Å away from the oxime oxygen atom (Figure 9). It is worth noting that about 19% (∼37%) of the HI-6 oxime is ionized in solution at the experimental pH of 7.0 (physiological pH of 7.4) according to the p*K*_a_ value of 7.63 for HI-6. However, the negatively charged oximate is not complementary to the AChE active site that has two anionic residues and 14 aromatic residues with pi-electrons. The p*K*_a_ of the oxime in this environment is presumably higher than in water. Only the neutral oxime gets into the active site and therefore AChE serves as a “buffer” that makes 100% of the oxime protonated. A tetrad is therefore needed to deprotonate the oxime hydrogen to increase the intrinsic nucleophilicity of HI-6. In addition to being a general base, the tetrad also assists in alignment of the oximate for attacking the OP moiety. Upon deprotonation, the oximate (RC = NO^-^) becomes less bulky than the neutral oxime (RC = NOH) and able to attack the phosphorus atom through a vacated narrow space that is in line with the O–P bond of Ser203 as shown in Figure 9 to form a pentavalent, trigonal bipyramidal structure at the transition state [5], [45]. The –P = O oxygen atom of the transition-state structure conceivably becomes an oxyanion that is stabilized by hydrogen bonds from Gly121, Gly122, and Ala204. At the transition state (Figure 9), the O^γ^ atom of Ser203 presumably forms a hydrogen bond with the imidazole proton as the distance between N^ε^ and O^γ^ is 3.1 Å in I3C. Finally at the product state, the oxyanion pushes the O^γ^ atom away to form a phosphonylated oxime and a reactivated AChE (Figure 9).

The reactivation mechanism of HI-6 proposed herein is consistent with a reactivation mechanism of ortho-7 conceivable from our previously reported ortho-7•tabun-*m*AChE crystal structure, wherein the oxime oxygen atom is 3.4-Å away from the His447 N^ε^ atom implying the ability to form a hydrogen bond between the two atoms [25]. According to the p*K*_a_ values of HI-6 and 1,6-hexylene-bis-*N*,*N*'-2-pyridiniumaldoxime described above, both HI-6•sarin^nonaged^-*m*AChE and ortho-7•tabun-*m*AChE structures suggest that the oxime group is protonated in the active site before reactivation and that the catalytic tetrad for HI-6 (Glu334-His447-H_2_O-Oxime) and the triad for ortho-7 (Glu334-His447-Oxime) serve as an effective base that deprotonates the oxime hydroxyl group to make it more nucleophilic to dephosphonylate the conjugated AChE than the catalytic serine residue to dephosphonylate the conjugated oxime. The structures disclosed herein and our previously reported ortho-7•tabun-*m*AChE structure provide, for the first time, plausible answers to long-standing questions with regard to (1) whether the oxime group is deprotonated or not in the AChE active site and (2) which active-site residue serves as a base to deprotonate the oxime group required for reactivation if the oxime is protonated. Furthermore, the HI-6•sarin^nonaged^-*m*AChE structure provides insights into reactivator design as discussed below.

### Insights into improved reactivator design

In I3Cr, the oxime oxygen atom is 6.5-Å away from the sarin phosphorus atom, and it does not form a hydrogen bond or a hydrogen-bond network to His447 (Figure 7A). In I3C, the same oxygen is 5.0-Å away and has a hydrogen-bond network to His447 (Figure 7B). Of 2,000 conformers collected at 50-ps intervals during the last 1.0-ns period of the 100 simulations, 235 (12%) and 14 (1%) conformers adopt the I3Cr- and I3C-like conformations, respectively. These percentages suggest that HI-6 converts from I3Cr to I3C at an approximate ratio of 12 to 1. Apparently, there is a significant energy barrier for the conversion from I3Cr to I3C or a significant potential energy difference between I3Cr and I3C, in addition to the energy barrier for changing from the Michaelis-Menten state to the transition state. The energy barrier or difference in potential energy between I3C and I3Cr likely limits the ability of HI-6 to reactivate sarin^nonaged^-*m*AChE, which probably makes the HI-6•sarin^nonaged^-*m*AChE crystal structure technically feasible. An important insight from this is to minimize the barrier or potential difference by modifying the chemical structure of HI-6. We previously reported an example of improving catalysis by reducing one energy barrier to the transition state [19], [42], which led to the development of a butyrylcholinesterase mutant as an effective cocaine hydrolase with clinical potential for treating cocaine overdose [43]. Herein we suggest that this type of modification offers a new venue to improved HI-6, although it may not be directly applicable to other conjugated AChEs.

### Structural difference between HI-6 in complex with conjugated and non-conjugated AChEs

In the HI-6•sarin^nonaged^-*m*AChE crystal structure aligned with the previously reported HI-6•*m*AChE crystal structure [24], the two proteins including all active-site residues except for Asp74 are nearly identical, and the carboxyamino-pyridinium portions of HI-6 in the two aligned complexes are almost the same as well. A marked difference between the two HI-6 molecules is at the oxime-pyridinium ring. The oxime oxygen atom has a hydrogen bond to N^Phe295^ in HI-6•*m*AChE, but it has no hydrogen bond or hydrogen-bond network to N^Phe295^ in HI-6•sarin^nonaged^-*m*AChE. In the two aligned complexes, the distance from the oxime oxygen atom of HI-6•*m*AChE to the phosphorus atom of HI-6•sarin^nonaged^-*m*AChE is 8.7 Å, while the corresponding distances in I3C and I3Cr are 5.0 and 6.5 Å, respectively. Consequently, no plausible reactivation mechanism could ever be drawn from the HI-6•*m*AChE structure, even with a sarin modeled into *m*AChE with the phosphorus atom 8.7-Å away from the oxime oxygen atom.

Furthermore, the difference between conjugated and non-conjugated AChE complexes suggests that caution should be used when extrapolating mechanistic information from the non-conjugated enzyme structure in the following examples. Visual inspection of the HI-6•*m*AChE crystal structure could lead to a hypothesis that the oxime group is deprotonated at the AChE active site before reactivation as there is no nearby residue that can deprotonate the oxime group. The pre-deprotonated oxime hypothesis is however unlikely given (1) that the p*K*_a_ value of HI-6 is 7.63, and (2) that effective reactivation requires higher nucleophilicity of a reactivator than that of the catalytic serine residue. In addition, the HI-6•*m*AChE structure could suggest that the hydrogen bond of HI-6 to N^Phe295^ hampers the conversion of HI-6 to the transition state thus reducing the reactivation efficiency. However, simulations presented herein shows that the oxime group does not form a hydrogen bond or a hydrogen-bond network to N^Phe295^ in the conjugated *m*AChE. Likewise, although the oxime-pyridinium of K027 has a bromide-mediated hydrogen bond to N^Phe295^ in the K027•*m*AChE crystal structure, it is premature to speculate that this hydrogen bond plays a role in reactivation efficiency or actually contributes to the lower potency of K027 than that of HI-6. This is because the hydrogen bond to N^Phe295^ could be absent in K027•sarin^nonaged^-*m*AChE based on the information garnered from this study. In the same vein, without the K027•sarin^nonaged^-*m*AChE structure, it is premature to assume that the bromide anion found at the Phe295 site entraps K027 even though the bromide has strong ionic interactions with the two cationic pyridinium rings.

### Conclusions

Despite the challenges in determining structures of intrinsically disordered reactivators in complex with enzymes possessing conjugates that are vulnerable to the reactivators and at the same time prone to develop resistance to reactivation, we have determined the HI-6•sarin^nonaged^-*m*AChE crystal structure and its computer model at the Michaelis-Menten state by combining crystallography and microsecond molecular dynamics simulation. In the HI-6•sarin^nonaged^-*m*AChE crystal structure, the carboxyamino-pyridinium portion of HI-6 is sandwiched by Tyr124 and Trp286, while the oxime-pyridinium portion is highly mobile and disordered. In the corresponding computed model (I3C) at the Michaelis-Menten state that was identified from the microsecond-scale MMDSs, the mobile oxime group forms a hydrogen-bond network to His447 with the oxime oxygen 5.0-Å away from the nonaged sarin phosphorus atom. Our studies suggest a reactivation mechanism of sarin^nonaged^-*m*AChE by HI-6 that is outlined as follows: (1) upon binding HI-6 adopts a populated conformation (I3Cr) and then converts to a less populated conformation (I3C) whose oxime oxygen atom has a hydrogen-bond network to His447 that simultaneously forms a hydrogen bond with Glu334 resulting in a tetrad of Glu334-His447-H_2_O-Oxime; (2) this tetrad not only positions the oxime oxygen atom 5.0-Å away from the sarin phosphorus atom but also deprotonates the oxime hydroxyl group; (3) upon deprotonation the oxyanion is less bulky and able to attack the phosphorus atom to form a pentavalent, trigonal bipyramidal structure at the transition state; (4) ejecting the O^γ^ atom of the catalytic Ser203 leading to the formation of a phosphonylated oxime and a reactivated AChE. Our studies also suggest that there is a significant energy barrier or potential energy difference between I3C and I3Cr. Structural modification of HI-6 to reduce the barrier/difference could lead to improved reactivators for sarin-conjugated mammalian AChEs.

## Materials and Methods

Sarin belongs to Schedule 1 Chemicals as defined in the Chemical Weapons Convention. Handling sarin requires suitable personal protection, training, and facilities, and is regulated by the Convention.

### Materials

Sarin was synthesized according to a reported method [46]. HI-6 and K027 were obtained from Drs. John Clement (Defense Research Establishment, Canada) and Kamil Kuca (University of Defence, Czech Republic), respectively.

### Cloning, expression, crystal screening, and generation of mAChE complexes

Cloning, expression, purification, and crystal screening of *m*AChE were performed as previously described [23]. A soaking solution (OX-buffer) containing HI-6 was prepared by dissolving approximately 10–20 mg of HI-6 into 500 µL X-buffer, which was composed of 28% (v/v) polyethylene glycol 750 monomethylether, 100 mM HEPES at a pH of 7.0, so that the concentration of HI-6 was much higher than the *K_D_* of HI-6 for sarin^nonaged^-*m*AChE. The stock solution was stored in liquid nitrogen and subsequently used for all soaking and control experiments. Crystals were first allowed to equilibrate slowly in X-buffer at 20°C. The X-buffer supplemented with 2 mM sarin was then added to the equilibrated crystals in ∼10 portions of 1 µL during a period of 5 minutes. Following inhibition of *m*AChE by sarin for 30 minutes, crystals were transferred to 2–4 µL of OX-buffer and incubated during a time ranging from one to ten minutes. The complex containing an aged form of sarin was generated in a similar fashion, with the exception that the crystal was incubated for 60 hours at 20°C, prior soaking with OX-buffer. As a reference, the aging half-life of sarin^nonaged^-AChE is ∼3 hours for the human enzyme at 37°C [8]. The HI-6•BR•*m*AChE complex was prepared by incubating a crystal of *m*AChE in X-buffer supplemented with 50 mM HI-6 and 1 M KBr for ∼30 minutes. The non-phosphonylated *m*AChE complex with K027 was generated as previously described for *m*AChE•oxime complexes [24]. All soakings were terminated by flash freezing of the crystal in liquid nitrogen.

### Collection, processing, and refinement of diffraction data

X-ray diffraction data were collected at the MAXlab synchrotron (Lund, Sweden), beam lines I911-2, I911-3 and I911-5 on MAR Research CCD detectors. Images were collected with an oscillation angle of 1.0° per exposure and the total oscillation range covered at least 140° per dataset. Intensity data were indexed and integrated with XDS [47] and scaled using Scala [48]. Seven datasets (soaking HI-6 for 1, 2, 3, 3, 4, 5, and 10 minutes) were collected for HI-6•sarin^nonaged^-*m*AChE, four datasets were collected for K027•*m*AChE, and three datasets were collected for HI-6•BR•*m*AChE. Anomalous data were collected after fluorescence scans around the bromide *K-*edge. The HI-6•sarin^nonaged^-*m*AChE structure obtained from the crystal with one-minute incubation with HI-6 was determined using rigid-body refinement with a modified *apo* structure of *m*AChE (PDB entry code: 1J06 [20]) as a starting model. To avoid model bias, Ser203, Trp286, and all small-molecule ligands were removed from the starting model. Further crystallographic refinement was carried out using restrained–isotropic–B-factor refinement as implemented in the program Refmac5 [49]. The refinement included 98% of the data while the remaining 2% was used to follow the progress of the refinement with R-free [50]. The R-free dataset was obtained from a previously determined structure (PDB entry code: 2GYU [23]) and was used for all datasets. Several rounds of refinement were performed, alternating with manual rebuilding of the model after visualization of 2|*F_o_*| – |*F_c_*| and |*F_o_*| – |*F_c_*| maps using programs O [51] and COOT [52]. Towards the end of the refinement, TLS refinement as implemented in phenix.refine was used [53]. Water molecules were added using COOT, phenix.refine and by manual model building using the 2|*F_o_*| – |*F_c_*| and |*F_o_*| – |*F_c_*| maps contoured at 1σ and 3.5σ, respectively. The remaining six datasets of HI-6•sarin^nonaged^-*m*AChE were partly refined by 20 cycles of rigid-body refinement and 20 cycles of restrained–isotropic–B-factor refinement using the modified *apo m*AChE structure as a starting model. The structures of HI-6•sarin^aged^-*m*AChE and K027•*m*AChE were determined and refined using the same approach as described for HI-6•sarin^nonaged^-*m*AChE. The angles and bonds for atoms bound to the phosphorous atom were determined using the Spartan program (Wavefunction Inc.) and the Hartree-Fock method with the 6-31G* basis set. The quality of the final models was evaluated using PROCHECK, WHATCHECK, and Rampage [54]–[56]. Figures were made using the PyMol program [57]. Atom ID definitions are shown in supporting information Figure S7.

### Site-directed mutagenesis

Recombinant “wild-type” *m*AChE was used as a template for site-directed mutagenesis introduced using the QuikChange II Site-Directed Mutagenesis Kit (Stratagene). All DNA sequences were verified by sequencing carried out at MWG Biotech (Germany). HEK-293F cells (Invitrogene) were maintained in Dulbecco's DMEM medium supplemented with 10% fetal calf serum and appropriate antibiotics. Cells (3×10^6^/100 mm plate) were transfected with 25 µg DNA-calcium phosphate co-precipitate (Invitrogene). After 24 hours, cells were washed and incubated with fresh media for additional 48 hours. Transfected clones were selected by incubation with media containing Geneticin (Invitrogene) at a concentration of 0.5 mg/mL until cell death subsided. Pools of stable clones were transferred to Dulbecco's DMEM medium supplemented with 2% fetal calf serum and the secreted *m*AChE mutant was collected in the supernatant.

### Determination of the Michaelis-Menten parameters of mAChE mutants

Enzyme activities of the secreted *m*AChE mutants described above were measured spectrophotometrically using a procedure based on the method developed by Ellman and co-workers [58]. This form of enzyme has the same catalytic parameters as the chromatographically purified enzyme used for crystallography according to our comparative kinetics studies. Acetylthiocholine iodide was used as a synthetic substrate that was hydrolyzed to produce thiocholine that in turn reacted with a 5,5′-dithiobis(2-nitrobenzoic acid) to yield a product measurable spectrophotometrically at 412 nm. To calculate the Michaelis-Menten constant (*K*_M_), 0.2 mM 5,5′-dithiobis(2-nitrobenzoic acid), 0.1 M phosphate buffer at pH of 8.0, and protein were mixed and the reaction was started by adding different concentrations of acetylthiocholine iodide and measured during 1 min in a BioTek Powerwave plate reader (yielding 8 replicates). All assays were performed at 30°C. The data were analyzed using the Enzyme Kinetics module of the program SigmaPlot 9.0.

### Determination of inhibition constants for oxime•mAChE complexes

Inhibition constants of oxime•*m*AChE complexes were obtained from the apparent Michaelis-Menten constant (*K*_M_^app^) and the apparent maximal velocity (*V^app^)* in the presence of HI-6 at various concentrations. The *K*_M_^app^ and *V^app^* values were determined using the Enzyme Kinetics module of SigmaPlot 9.0. The mode of inhibition was determined by analysis of Lineweaver-Burke plots using the statistical module of SigmaPlot. The best-fit results were obtained by using a mixed partial inhibition model (see supporting information Figure S5). At least four replicates were measured for each HI-6 concentration.

### Reactivation kinetics

Reactivation kinetics was investigated using the reported discontinuous method [8]. To investigate the reactivation rate under the buffer condition that was similar to the one used during crystal soaking, the sarin^nonaged^-*m*AChE was incubated with HI-6 at various concentrations (20–300 µM) in a buffer composed of 32% PEG 750 MME, 100 mM HEPES at pH of 7.0, 25 mM NaCl, at 25°C. After 3, 6, 9, 12, and 15 minutes, samples were withdrawn and the activity was measured as described above. Secondary plots of *k_obs_* versus the concentration of HI-6 (supporting information Figure S6) were used to determine the affinity between HI-6 and the phosphonylated enzyme (*K_D_*) and the rate constant for the displacement of the phosphorous conjugate (*k_r_)*. The reactivation of diisopropyl fluorophosphate- (DFP-) or sarin-inhibited recombinant human AChE (*h*AChE) by K027 was measured using a reported buffer condition [8]. The use of *h*AChE was for comparison with literature data. The concentrations of K027 used in the reactivation of the sarin^nonaged^-inhibited *h*AChE and the DFP-inhibited *h*AChE were 0.1–75 µM and 25–3000 µM, respectively.

### Determination of the apparent pK_a_ of HI-6

The apparent p*K*_a_ of HI-6 was determined by the ^1^H NMR chemical shift change of protons of HI-6 specified in supporting information Figure S4 as a function of solution pH. Thus, ^1^H NMR spectra of 13 solutions with 20 mM HI-6 and 200 mM phosphate buffer in D_2_O at pH of 5.58–9.86 were recorded. Proton chemical shifts were referenced to internal 3-(trimethylsilyl)propionic acid(*d*_4_). The apparent p*K*_a_ of HI-6 was obtained by non-linear least squares fit of the observed chemical shifts to Equation 1 by applying the Levenberg-Marquardt method. Parameters δ_HA_, δ_A_, and p*K*_a_ were defined as adjustable.
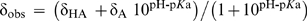
(1)where 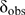 is the observed ^1^H NMR chemical shift at a defined pH,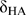 is the limiting ^1^H NMR chemical shift of the acid form and 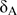 is the limiting ^1^H NMR chemical shift of the anionic form.

### Microsecond molecular dynamics simulations

MMDSs were performed by using the PMEMD module of AMBER 8.0 [59] with the AMBER force field (frcmod.ff99) [60]. The topology and coordinate files were generated by the PREP, LINK, EDIT, and PARM modules of AMBER 5.0 [61]. All simulations used (1) a dielectric constant of 1.0, (2) the Berendsen coupling algorithm [62], (3) a periodic boundary condition at a constant temperature of 300 K and a constant pressure of 1 atm with isotropic molecule-based scaling, (4) the Particle Mesh Ewald method to calculate long-range electrostatic interactions [63], (5) a time step of 1.0 fs, (6) the SHAKE-bond-length constraints applied to all the bonds involving the H atom, (7) saving the image closest to the middle of the “primary box” to the restart and trajectory files, (8) formatted restart file, and (9) default values of all other inputs of the PMEMD module.

The atomic charges of the sarin^nonaged^-conjugated serine residue and HI-6 with a protonated oxime group instead of an oxime anion were obtained according to the RESP procedure [64] with *ab initio* calculations at the HF/6-31G*//HF/6-31G* level using Gaussian98 [65]. The starting structure of HI-6•sarin^nonaged^-*m*AChE was taken and modified from an early crystal structure that was partially refined from the dataset with 1-minute exposure to HI-6. This crystal structure contains residues 493–496 (which were omitted in the final crystal structure) and has the oxime oxygen atom modeled to form a hydrogen bond to the main-chain nitrogen atom of Phe295 (N^Phe295^). The modification was to rotate torsions along -N-CH_2_-O- of HI-6 in order to place the oxime oxygen atom 3.7 Å and 7.2 Å away from the phosphorus atom and from N^Phe295^, respectively. This modification was for investigating the oxime hydrogen bond to N^Phe295^. For *m*AChE, His447 was treated as HID; His223 and His387 were treated as HIE; all other His residues were treated as HIP. A total of 54 crystallographically determined water molecules (named HOH) that were located inside the enzyme were included in the *m*AChE structure for simulations.

The water-containing HI-6•sarin^nonaged^-*m*AChE complex was refined by energy minimization using a dielectric constant of 1.0 and 100 cycles of steepest-descent minimization followed by 100 cycles of conjugate-gradient minimization. The resulting complex was solvated with 16,929 TIP3P water molecules (named WAT) [66], leading to a system of 59,265 atoms. The WAT molecules were obtained from solvating the complex using a pre-equilibrated box of 216,000 TIP3P molecules, whose hydrogen atom charge was set to 0.4170, where any water molecule was removed if it had an oxygen atom closer than 2.2 Å to any solute atom or a hydrogen atom closer than 2.0 Å to any solute atom, or if it was located further than 8.2 Å along the x-, y-, or z-axis from any solute atom.

The solvated complex system were energy-minimized for 100 cycles of steepest-descent minimization followed by 100 cycles of conjugate-gradient minimization to remove close van der Waals contacts in the system, then heated from 0 to 300 K at a rate of 10 K/ps under constant temperature and volume, and finally simulated at 300 K under constant temperature and pressure. One hundred 10-ns-long simulations were carried out on 200 Apple Xservers each equipped with two G5 processors at a clock rate of 2.0/2.3 GHz, and each of these simulations used a unique seed number for initial velocities.

Average structures were obtained by using the CARNAL module of AMBER 5.0. Cluster analyses were performed by using the PTRAJ module [35] of AMBER 10. RMSDs were calculated by using the McLachlan algorithm [67] as implemented in ProFit V2.6 (http://www.bioinf.org.uk/software/profit/). The C&F RMSDs were obtained by (1) generating symmetry mates within 12 Å using PyMol 0.99rc6, (2) identifying not-free residues that have a distance of <8.0 Å between any non-hydrogen atom of the structure in the primary cell and any non-hydrogen atom of the symmetry mates, (3) identifying hot residues that have alpha-carbon atoms with B factors greater than the average alpha-carbon B factor, (4) identifying short peptides with up to four residues that are between two hot residues, two not-free residues, or between a hot and a not-free residue, (5) deleting the hot and not-free residues and the short peptides from the crystal structure and from the structure to be compared, and (6) computing the alpha-carbon RMSD of the truncated proteins using ProFit. The coordinates of four MMDS-generated structures are provided in supporting information Datasets S1, S2, S3 and S4.

## Supporting Information

Figure S1 Cross-eyed stereo view of HI-6•sarin^nonaged^-*m*AChE.
(3.99 MB TIF)

Figure S2 Omit electron density map covering HI-6•sarin^nonaged^-*m*AChE, calculated after simulated annealing of a model in which HI-6, Asp74, sarin^nonaged^-Ser203, and Trp286 were omitted. The |*Fo*| - |*Fc*| map is contoured at 3σ (green) and -3σ (red).
(7.30 MB TIF)

Figure S3 Superposition of HI-6•sarin^nonaged^-*m*AChE (yellow) and HI-6•sarin^aged^-*m*AChE (green) with the |*Fo*| - |*Fc*| electron density map of HI-6•sarin^nonaged^-*m*AChE contoured at 3σ.
(4.12 MB DOC)

Figure S4 ^1^H NMR chemical shift of proton β *versus* pH from experiment and regression model.
(1.02 MB TIF)

Figure S5 Hydrolysis of acetylthiocholine by wild-type *m*AChE in the presence of different concentrations of HI-6 (A) and the resulting Lineweaver-Burke plot (B). The partial mixed inhibition model employed for the analysis of HI-6 binding to *m*AChE substitutions (C). Letters E, S, P, and I designate the free enzyme, the substrate, the product, and the inhibitor HI-6, respectively.
(0.84 MB TIF)

Figure S6 Primary (A) and secondary (B) plots of reactivation of the DFP-inhibited *h*AChE by K027.
(0.94 MB TIF)

Figure S7 Compounds and atom ID definitions used in this study.
(0.76 MB TIF)

Dataset S1 A2C
(0.47 MB DOC)

Dataset S2 A3C
(0.47 MB DOC)

Dataset S3 I3Cr
(4.50 MB DOC)

Dataset S4 I3C
(4.50 MB DOC)
