# Supplementary figures and images for: Structure of HI-6•Sarin-Acetylcholinesterase Determined by X-Ray Crystallography and Molecular Dynamics Simulation: Reactivator Mechanism and Design
Source: PLoS One. 2009 Jun 18;4(6):e5957. doi: 10.1371/journal.pone.0005957 (PMC2693926; doi:10.1371/journal.pone.0005957)

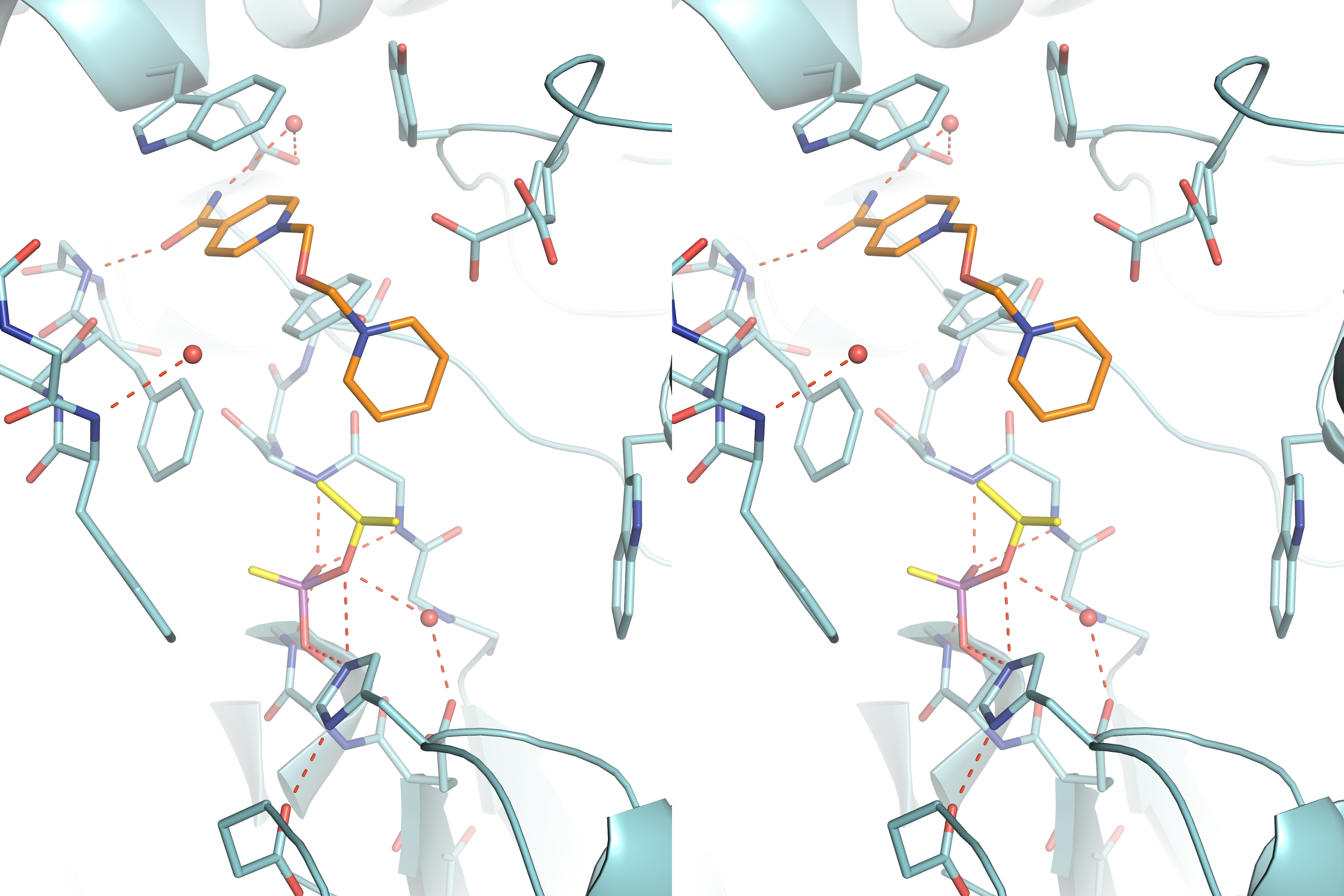

Supplement: Figure S1 — Cross-eyed stereo view of HI-6•sarinnonaged-mAChE. (3.99 MB TIF) [file pone.0005957.s001.tif]

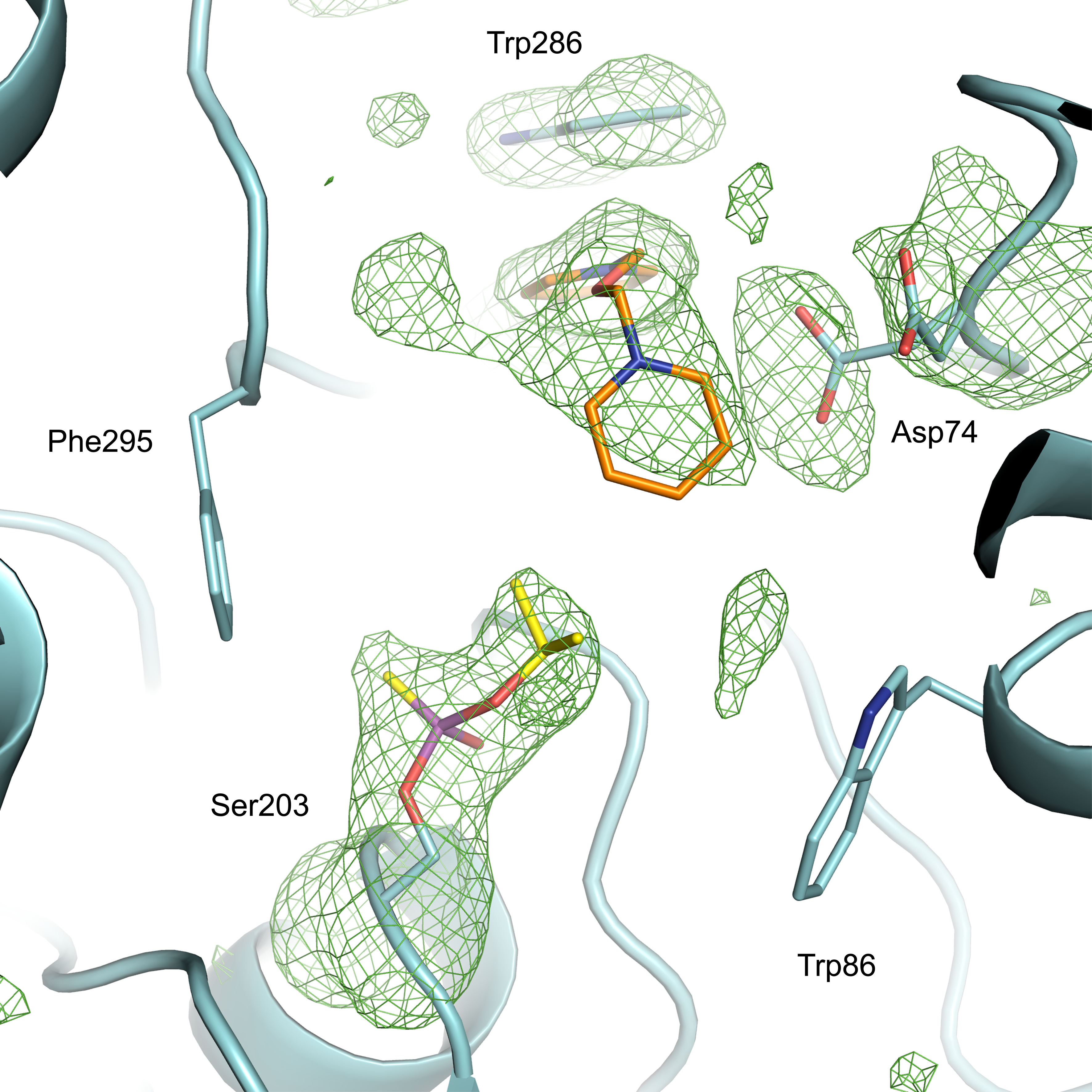

Supplement: Figure S2 — Omit electron density map covering HI-6•sarinnonaged-mAChE, calculated after simulated annealing of a model in which HI-6, Asp74, sarinnonaged-Ser203, and Trp286 were omitted. The |Fo| - |Fc| map is contoured at 3σ (green) and -3σ (red). (7.30 MB TIF) [file pone.0005957.s002.tif]

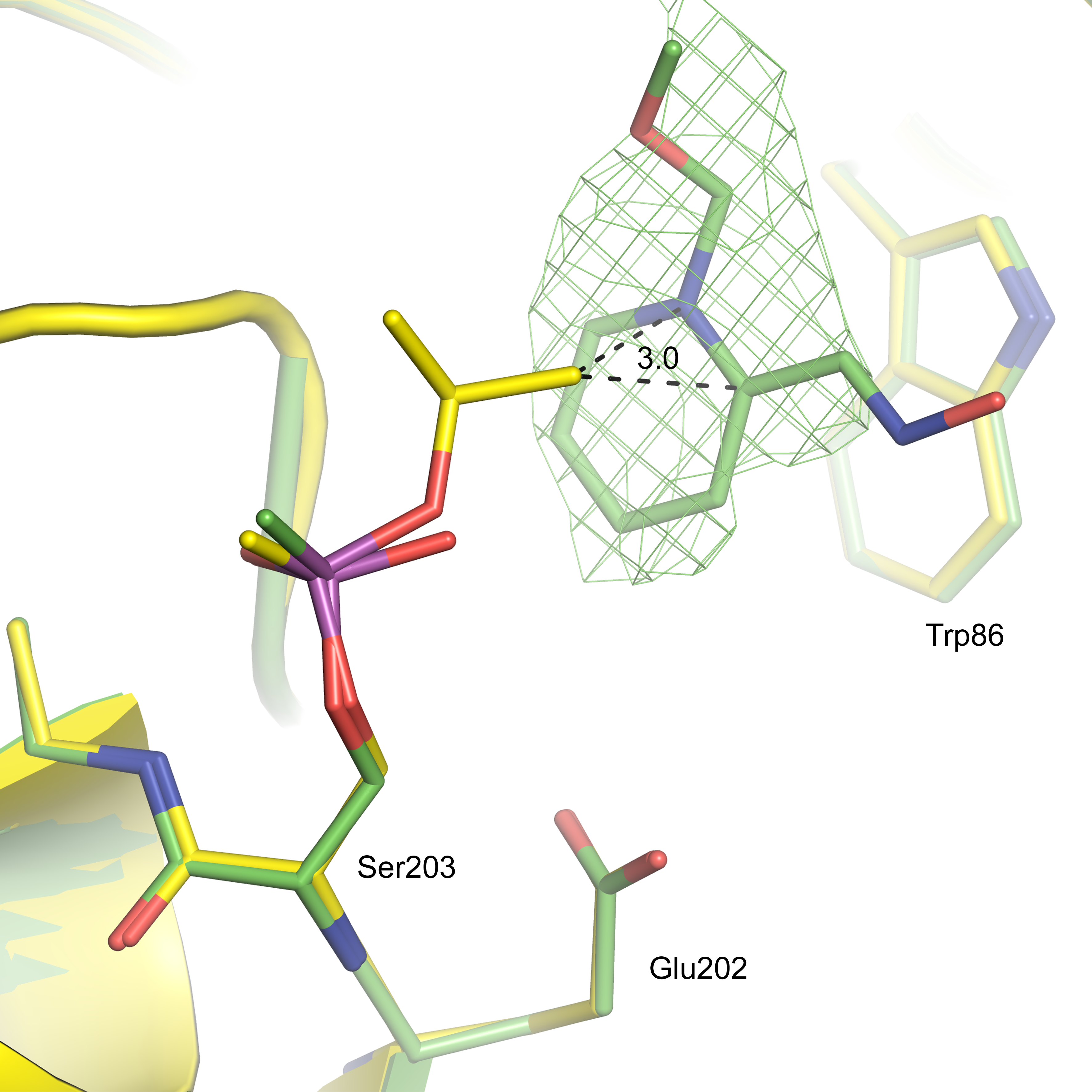

Supplement: Figure S3 — Superposition of HI-6•sarinnonaged-mAChE (yellow) and HI-6•sarinaged-mAChE (green) with the |Fo| - |Fc| electron density map of HI-6•sarinnonaged-mAChE contoured at 3σ. (4.12 MB DOC) [file pone.0005957.s003.doc]

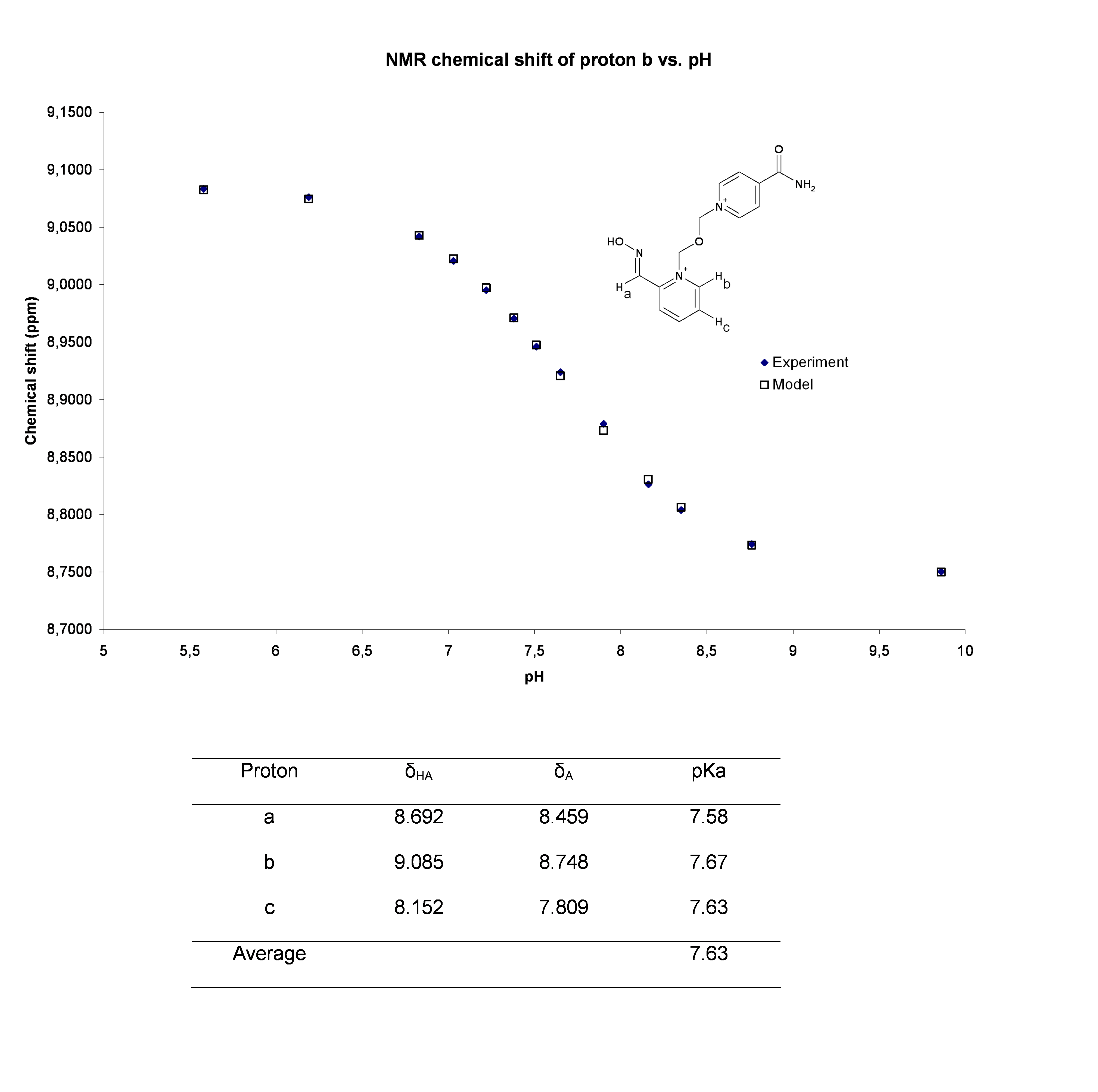

Supplement: Figure S4 — 1H NMR chemical shift of proton β versus pH from experiment and regression model. (1.02 MB TIF) [file pone.0005957.s004.tif]

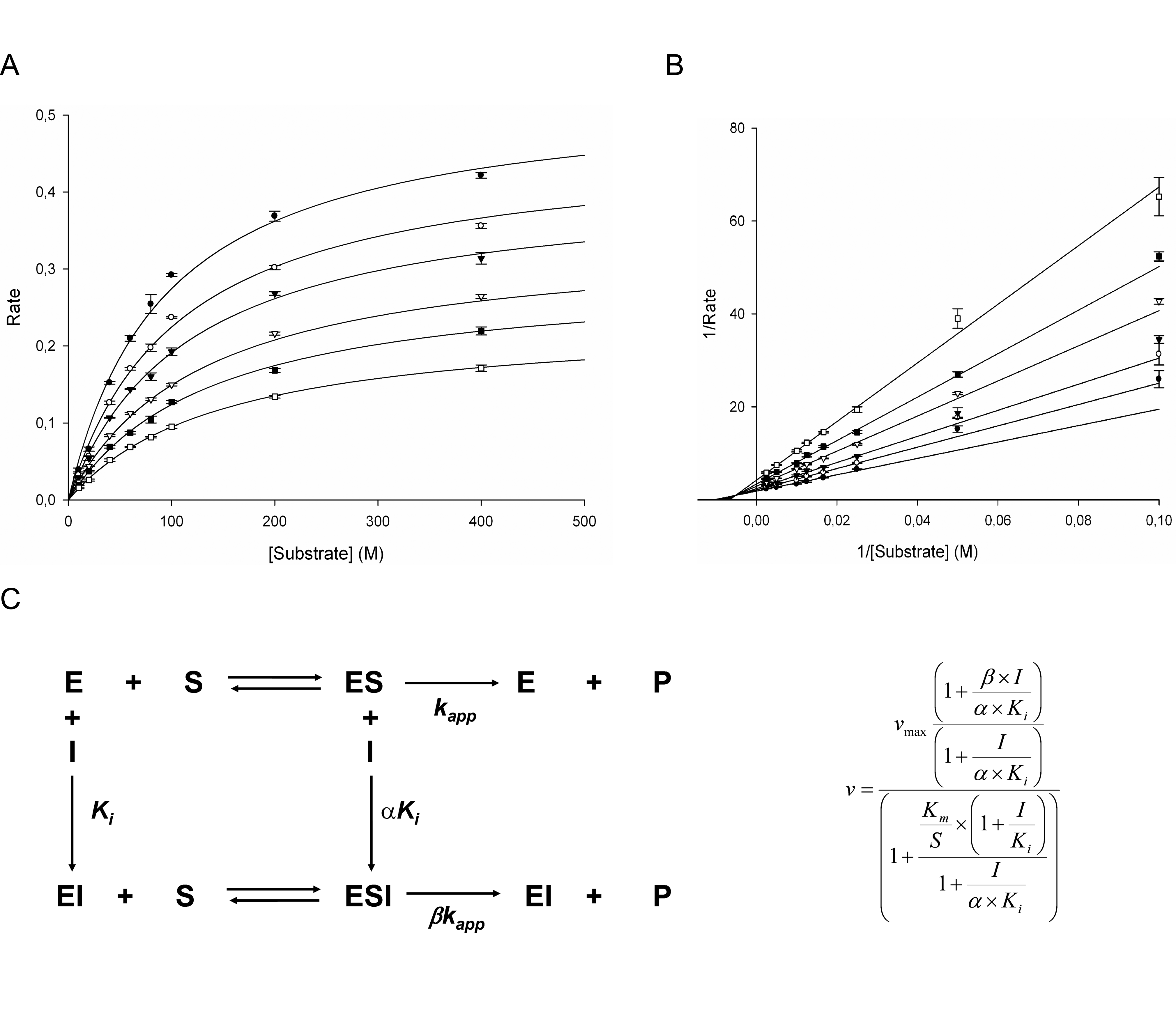

Supplement: Figure S5 — Hydrolysis of acetylthiocholine by wild-type mAChE in the presence of different concentrations of HI-6 (A) and the resulting Lineweaver-Burke plot (B). The partial mixed inhibition model employed for the analysis of HI-6 binding to mAChE substitutions (C). Letters E, S, P, and I designate the free enzyme, the substrate, the product, and the inhibitor HI-6, respectively. (0.84 MB TIF) [file pone.0005957.s005.tif]

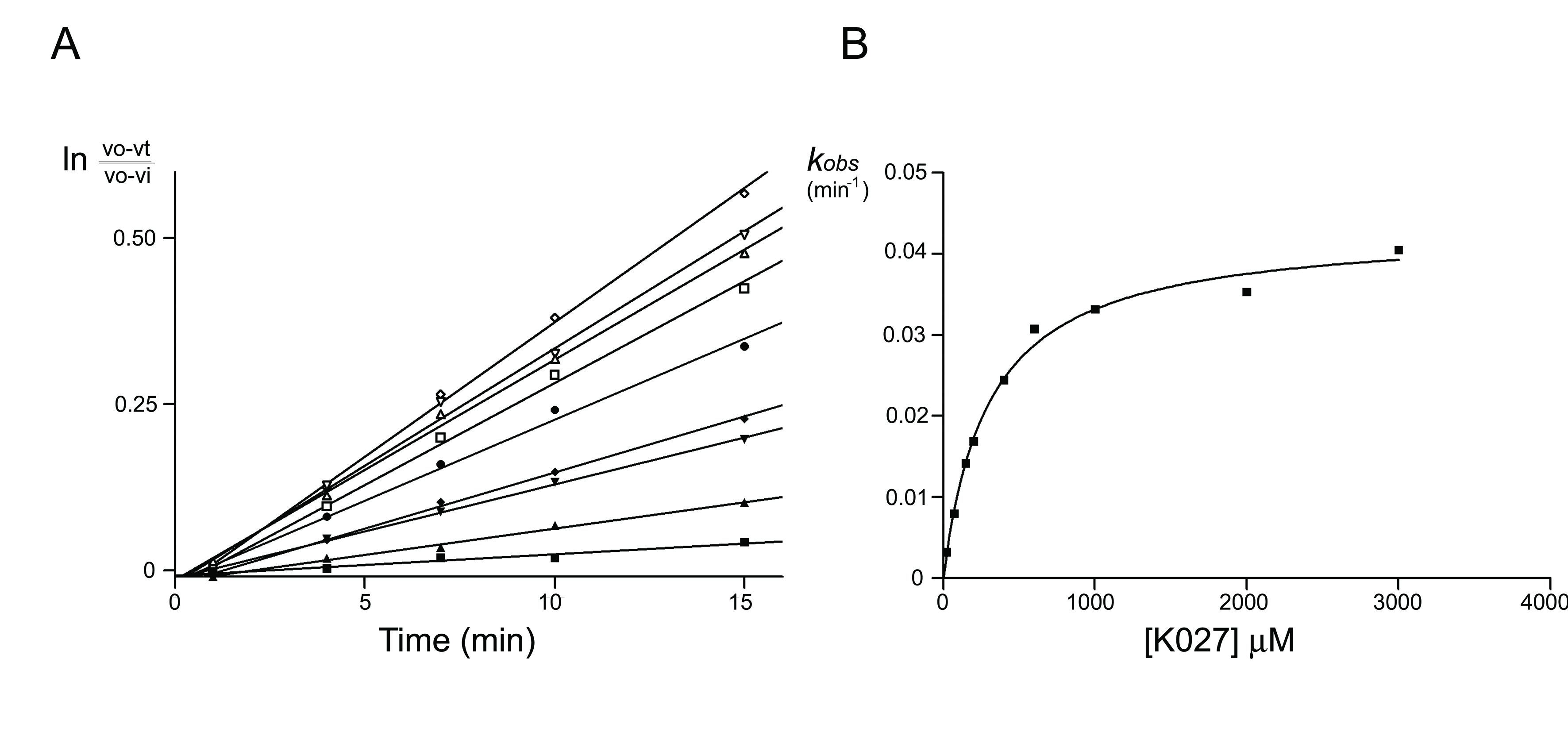

Supplement: Figure S6 — Primary (A) and secondary (B) plots of reactivation of the DFP-inhibited hAChE by K027. (0.94 MB TIF) [file pone.0005957.s006.tif]

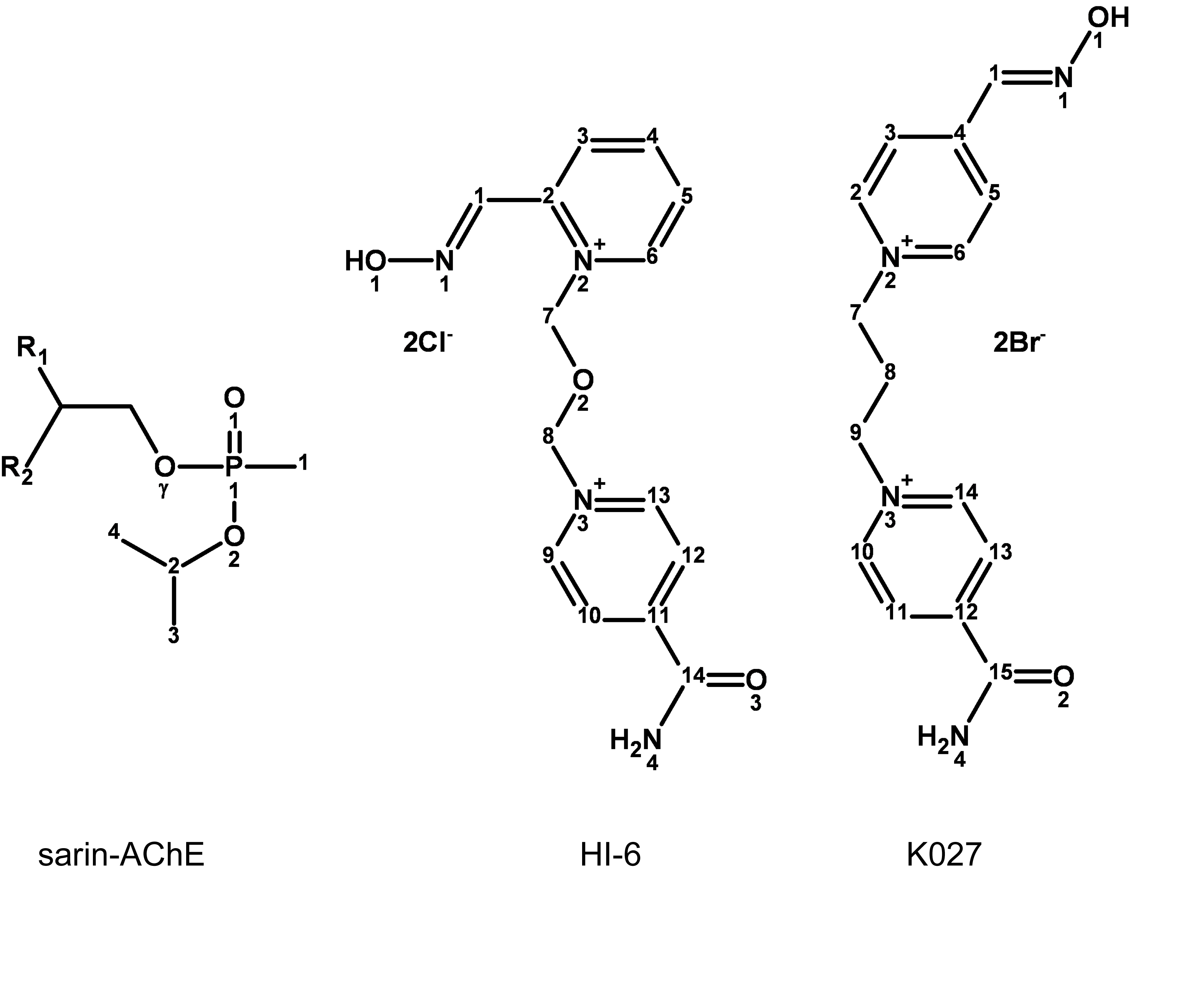

Supplement: Figure S7 — Compounds and atom ID definitions used in this study. (0.76 MB TIF) [file pone.0005957.s007.tif]
